# Acetate derived from the intestinal tract has a critical role in maintaining skeletal muscle mass and strength in mice

**DOI:** 10.14814/phy2.16047

**Published:** 2024-06-04

**Authors:** Saki Kobayashi, Katsutaro Morino, Takuya Okamoto, Mitsumi Tanaka, Shogo Ida, Natsuko Ohashi, Koichiro Murata, Tsuyoshi Yanagimachi, Juro Sakai, Hiroshi Maegawa, Yukihiro Fujita, Shinji Kume

**Affiliations:** ^1^ Division of Endocrinology and Metabolism, Department of Medicine Shiga University of Medical Science Otsu Japan; ^2^ Institutional Research Office, Shiga University of Medical Science Otsu Japan; ^3^ CMIC Pharma Science Nishiwaki Japan; ^4^ Division of Molecular Physiology and Metabolism Tohoku University Graduate School of Medicine Sendai Japan; ^5^ Division of Metabolic Medicine, Research Center for Advanced Science and Technology The University of Tokyo Tokyo Japan; ^6^ Present address: Department of Diabetes and Endocrine Medicine Kagoshima University Graduate School of Medical and Dental Sciences Kagoshima‐city Japan; ^7^ Present address: Department of Endocrinology and Metabolism, Graduate School of Medicine Hirosaki University Hirosaki‐sity Japan; ^8^ Present address: Yasu City Hospital Yasu‐city Japan

**Keywords:** acetate, microbiome, short‐chain fatty acid, skeletal muscle

## Abstract

Acetate is a short‐chain fatty acid (SCFA) that is produced by microbiota in the intestinal tract. It is an important nutrient for the intestinal epithelium, but also has a high plasma concentration and is used in the various tissues. Acetate is involved in endurance exercise, but its role in resistance exercise remains unclear. To investigate this, mice were administered either multiple antibiotics with and without oral acetate supplementation or fed a low‐fiber diet. Antibiotic treatment for 2 weeks significantly reduced grip strength and the cross‐sectional area (CSA) of muscle fiber compared with the control group. Intestinal concentrations of SCFAs were reduced in the antibiotic‐treated group. Oral administration of acetate with antibiotics prevented antibiotic‐induced weakness of skeletal muscle and reduced CSA of muscle fiber. Similarly, a low‐fiber diet for 1 year significantly reduced the CSA of muscle fiber and fecal and plasma acetate concentrations. To investigate the role of acetate as an energy source, acetyl‐CoA synthase 2 knockout mice were used. These mice had a shorter lifespan, reduced skeletal muscle mass and smaller CSA of muscle fiber than their wild type littermates. In conclusion, acetate derived from the intestinal microbiome can contribute to maintaining skeletal muscle performance.

## 1 INTRODUCTION

Aging‐related loss of skeletal muscle mass (sarcopenia) and muscle strength (dynapenia) are not simply physiological changes but also affect quality of life and life expectancy (Bernabeu‐Wittel et al., 2019; Cruz‐Jentoft et al., 2019). These effects are more pronounced in individuals with cancer or chronic diseases than in healthy individuals and have become a major social problem in aging populations (Yuan & Larsson, 2023). Frailty is a multifactorial geriatric syndrome characterized by decreased reserve and diminished resistance to stressors (Dent et al., 2019). Sarcopenia and dynapenia are major components of frailty characterized by weight loss, muscle weakness, exhaustion, slow walking speed, and low physical activity (Fried et al., 2001). The biological process during sarcopenia is characterized by a loss of motor neurons and capillarization, mitochondrial dysfunction, and satellite cell dysfunction (Dent et al., 2019; Larsson et al., 2019).

The microbiome is closely related to human health and disease (Sekirov et al., 2010). Over the past decade, there has been a growing understanding of the relationship between the intestinal environment and systemic disease (Hou et al., 2022). A fiber‐rich diet promotes the production of metabolites such as short‐chain fatty acids (SCFAs), secondary bile acids, and some amino acids that regulate homeostasis in skeletal muscle by improving insulin sensitivity (Kreznar et al., 2017). In addition, systemic inflammation is induced by bacterial endotoxins such as lipopolysaccharides (Cristofori et al., 2021). Recent human studies have revealed an association between microbiota and sarcopenia (Liu et al., 2021). Moreover, several epidemiological studies have reported a positive association between higher fiber intake and skeletal mass (Frampton et al., 2021; Takahashi et al., 2022) or physical performance (Frampton et al., 2021). In the gut, microbiota metabolize dietary fiber by fermentation, producing SCFAs, including acetate, propionate, and butyrate (Lemecha et al., 2018; Lu et al., 2016; Luo et al., 2017). Acetate is a metabolic substrate, which is used in fatty acid synthesis or in the tricarboxylic acid cycle through acetyl‐CoA synthetase 2 (AceCS2). However, the roles of the intestinal microbiota and acetate in the maintenance of skeletal muscle mass and strength are unclear. In this mouse study, the role of acetate was investigated to elucidate the relationship between dietary fiber and skeletal muscle mass and strength.

## 2 MATERIALS AND METHODS

### 2.1 Animals and diet groups

All mice were maintained on a 12 h/12 h light/dark cycle (lights on at 8 a.m.) under constant temperature (25°C), with unlimited access to food and water. For the antibiotics experiment, C57BL/6 mice at 10 weeks of age were allocated to either an antibiotic treatment group (Abx+) for 2 weeks or a control group (Abx‐) without antibiotics fed with a standard diet (CE‐2, CLEA Japan, Tokyo, Japan). We used an antibiotic treatment as reported previously (Okamoto et al., 2019). The chemicals and reagents used were sodium acetate from Sigma (S5636), streptomycin and penicillin (Meiji Seika Pharma Co., Ltd., Tokyo, Japan), and vancomycin hydrochloride (V0252), metronidazole (M1977), ciprofloxacin (C3262), ceftazidime hydrate (C1635), gentamycin sulfate (G1658), and neomycin sulfate (N1755) from LKT laboratories, Inc. (St Paul, MN, USA) as described previously (Okamoto et al., 2019). For oral acetate supplementation, an antibiotic treatment with a 150 mM sodium acetate solution (Abx + acetate) was added (Smith et al., 2013). For the low‐fiber diet (LFD) experiments, C57BL/6 mice at 10 weeks of age were fed either an LFD (D20011402, Research Diets Inc., New Brunswick, NJ, USA) (Matt et al., 2018) or a standard diet (CE‐2, CLEA Japan). Table S1 (https://doi.org/10.6084/m9.figshare.25672320.v1).

Acetyl‐coenzyme A synthetase 2‐like, mitochondrial knockout mice (AceCS2‐KO) were generated as previously described (Sakakibara et al., 2009). We conducted all experiments on male mice, which were considered complete knockout mice. The mice were fed a standard diet (CLEA Japan) until the age of 64 weeks.

Animal handling and experimentation were conducted in accordance with the guidelines of the Research Center for Animal Life Science at Shiga University of Medical Science (Approved number #29‐23, #3‐41). All experimental protocols were approved by the Gene Recombination Experiment Safety Committee and the Research Center for Animal Life Science at Shiga University of Medical Science (Approved number #2020‐4‐4, 2022‐7‐4, 2023‐6‐1).

### 2.2 Tissue collection

Mice were euthanized by cervical dislocation with sevoflurane inhalation for tissue collection. Epididymal white adipose tissue (WAT) and skeletal muscles were immediately dissected, snap‐frozen in liquid nitrogen, and stored at −80°C until analysis. Blood samples were collected from the inferior vena cava into heparinized tubes and centrifuged at 1000 × ***g*** for 15 min. The derived plasma was stored at −80°C until further analysis. For antibiotic treatment experiments, mice were fasted for 12 h prior to tissue collection. All other mice were sacrificed at ad libitum feeding conditions.

### 2.3 Grip strength measurement

Grip strength of both forelimbs was measured using a MK‐380Si grip strength meter (Muromachi Kikai Co. Ltd, Tokyo, Japan) as previously described (Aartsma‐Rus & van Putten, 2014). The forelimbs were chosen because there is less variation in the grip strength values than in the four limb measurements. Three measurements were recorded and the values were averaged (Ono et al., 2020). To measure endurance of grip strength, the grip strength test was repeated fifteen times under ad libitum feeding conditions (Takeshita et al., 2017).

### 2.4 Measurement of SCFAs

SCFAs in plasma and cecum were measured as described previously (Okamoto et al., 2019). In brief, 20 μL of plasma or ~100 mg of feces were treated with ethanol/water (3:7, vol/vol), which was added at room temperature. Extracted samples were quantified using liquid chromatography–mass spectrometry(MS)/MS. Liquid chromatography was performed using an Acquity UPLC system (Waters, Milford, MA, USA) and an analytical column (Acquity HSS T3 2.1 × 150 mm, 1.8 μm; Waters). For mass detection, an API4000 tandem mass spectrometer (AB Sciex, Foster City, CA, USA) was used.

### 2.5 Fecal specimen collection, culture, and DNA extraction

In all experiments, fecal samples were collected from cecum under fed conditions and were immediately frozen at −80°C. The samples were freeze‐dried using VD‐250R (Taitec) and then powdered using a multi‐bead shocker (MB‐3000, Yasui Instruments) at 1500 rpm for 2 min. Lysis Solution F (318–06271, Nippon Gene) was added to the crushed sample and allowed to stand at 65°C for 10 min. The supernatant was then centrifuged at 12,000 × ***g*** for 2 min and the supernatant was separated. DNA was extracted from the samples using a DNA Extraction Kit (Lab‐Aid 824 s, ZEESAN). The concentrations of the extracted DNA solutions were measured using Synergy LX (Agilent Technologies) and the QuantiFluor dsDNA System (E2670, Promega).

### 2.6 16S/rRNA analysis

Universal bacterial 16S rRNA sequencing was used to quantify the sizes of bacterial populations as reported previously (Okamoto et al., 2019).

### 2.7 Taxonomic and ecological analyses

For read quality filtering, we used the Fastx toolkit version 0.0.14 and the Fastx barcode splitter tool to extract only the sequences that had a start sequence exactly matching that of the primer used. The primer sequence of the extracted sequence was deleted. After that, we removed the sequences with quality values <20 using Sickle tools and discarded sequences of ≤130 bases and their paired sequences. For merge reads, we merged arrays that passed the quality filtering criteria using the paired‐end merge script FLASH (ver. 1.2.11). The condition of merging was set to a fragment length of 410 bases after merging, a fragment length of read of 280 bases, and a minimum overlap length of 10 bases. After removing chimeric and noisy sequences with the dada2 plugin of Qiime2 (ver. 2023.5), representative sequences and ASV tables were output. Using the feature‐classifier plugin, we compared the obtained representative sequences with the 97% OTU of Greengene (ver. 13_8) and inferred phylogeny. Alignment and phylogeny plug‐ins were used to create the phylogenetic tree. Microbial diversity was measured using the Shannon index (richness and evenness) and Chao1 index (richness), which are alpha‐diversity indexes.

### 2.8 Total RNA preparation and reverse transcription quantitative PCR analysis

Total RNA was extracted from tissues using the RNeasy Mini Kit (Cat. No. 74106, Qiagen Inc., Valencia, CA, USA). The cDNA was synthesized using reverse transcription reagents (R007B, Takara Bio, Otsu, Japan). Transcript abundance was assessed by real‐time quantitative PCR on an Applied Biosystems 7500 Real‐Time PCR System (Thermo Fisher Scientific K.K., Yokohama, Japan) with SYBR Green (SCR_008426, Bio‐Rad Laboratories, Hercules, CA, USA). Analytic data were adjusted with GAPDH mRNA expression as an internal control. Primer sequences are provided in the Table S2 (https://doi.org/10.6084/m9.figshare.25672338.v1).

### 2.9 Histological analysis and morphometry

For fluorescent imaging, dissected tibialis anterior muscles were mounted on cork disks using tragacanth gum before snap freezing in liquid‐nitrogen‐cooled isopentane. Next, 5 μm transverse sections were cryosectioned and fixed for 15 min with 4%‐paraformaldehyde phosphate buffer solution (09154–56, Nacalai tesque, Kyoto, Japan). For 15 min, sections were permeabilized with 0.1% Triton X‐100 in DPBS and 0.1% polyoxyethylene sorbitan monolaurate. Then the sections were nuclease tested in DPBS for 30 min prior to staining overnight at 4°C with anti‐laminin antibody (ab11575, Abcam). Next day, Alexa Fluor594 (A‐11037, Invitrogen, Tokyo, Japan) was used. Images were visualized under a fluorescent microscope (BZ‐X710, Keyence, Osaka, Japan) with a replaced objective lens (CFI Plan Apochromat λ 4×,10x,20x Nikon Instruments Inc., Tokyo, Japan) and used its software BZ‐analyzer (Keyence) as previously described (Kondo et al., 2018). The fluorescence microscope was equipped with a motorized stage and a CCD camera with the dynamic range of 14 bit, and excitation light emission was kept at the minimal level (low‐photobleach mode). The filters included BZ‐X filter GFP (OP‐87763, Keyence), BZ‐X filter Cy5 (OP‐87766, Keyence), BZ‐X filter TRITC (OP‐87764, Keyence), ET‐Narrow Band EGFP to minimize autofluorescence (49,020, Chroma Technology, Brattleboro, VT, USA), ET‐Cy5 narrow excitation (49,009, Chroma), and ET‐Gold FISH (49,304, Chroma). The cross‐sectional area (CSA) of muscle fibers in the tibialis anterior muscle were measured using a hybrid cell count application (Keyence) as previously described (Uezumi et al., 2021). All fibers were counted in samples that contained 318–1977 fibers/section. Fibers of 100–7446 μm^2^ were chosen for further analysis.

### 2.10 Statistical analyses

All quantitative data except for probability of survival are expressed as mean ± SD, and probability of survival data are expressed using Kaplan–Meier estimates. Student's *t*‐test was used to evaluate differences between two groups, and one‐way analysis of variance and subsequent post hoc Tukey tests were used to determine the significance of differences where multiple comparisons were required. Spline curves with 95% confidence intervals were generated for evaluating trends in grip strength endurance. Data were analyzed using commercial software (Prism 10: GraphPad, San Diego, CA, USA; JMP Pro18: SAS institute Japan, Tokyo, Japan; Exploratory ver. 8.4: Exploratory Inc., Mill Valley, USA). *p* < 0.05 was considered to represent statistical significance.

## 3 RESULTS

### 3.1 Oral administration of antibiotics reduces grip strength in mice

**FIGURE 1:**
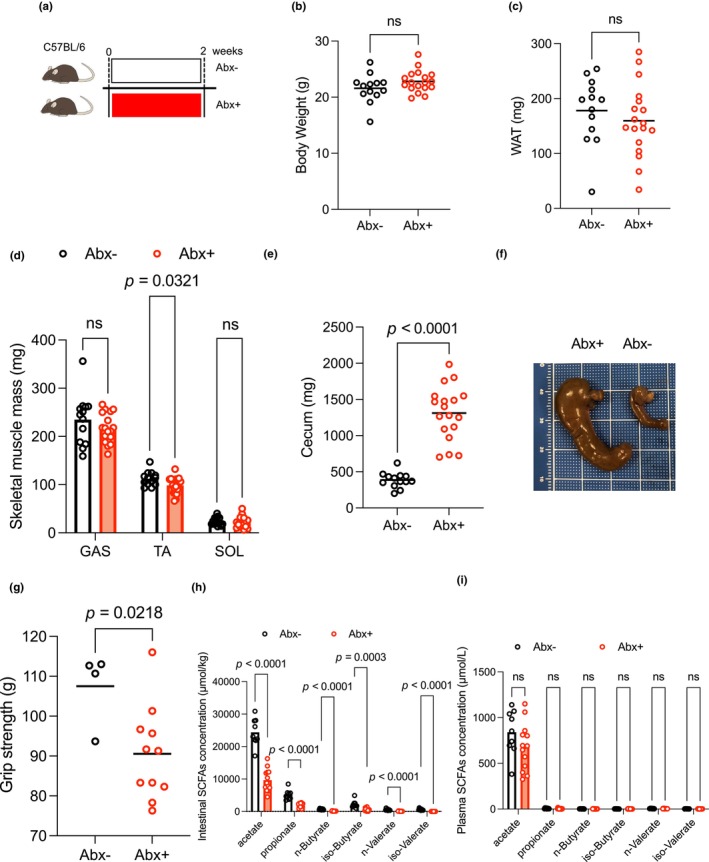
Antibiotic treatment for 2 weeks reduced grip strength in mice. (a) Experimental protocol. C57BL/6 mice were allocated to two groups and administered antibiotics (100 μg/mL neomycin, 50 μg/mL streptomycin, 100 U/mL penicillin, 50 μg/mL vancomycin, 100 μg/mL metronidazole, 125 μg/mL ciprofloxacin, 100 μg/mL ceftazidime, and 170 μg/mL gentamicin in their drinking water) or not for 2 weeks (Abx‐, *n* = 13; Abx+, *n* = 18). (b) The bodyweight of Abx‐ and Abx + mice (Abx‐, *n* = 13; Abx+, *n* = 18). (c) WAT mass of Abx‐ and Abx + mice (Abx‐, *n* = 13; Abx+, *n* = 18). (d) Skeletal muscle masses of Abx‐ and Abx + mice (Abx‐, *n* = 13; Abx+, *n* = 18). (e and f) Cecum of Abx‐ and Abx + mice (Abx‐, *n* = 13; Abx+, *n* = 18). (g) Grip strength of Abx‐ and Abx + mice (Abx‐, *n* = 4; Abx+, *n* = 11). (h) Intestinal SCFA concentrations of Abx‐ and Abx + mice (Abx‐, *n* = 9; Abx+, *n* = 13). (I) Plasma SCFA concentration of Abx‐ and Abx + mice (Abx‐, *n* = 9; Abx+, *n* = 13). Data expressed as mean; ns, not statistically significant. The lines indicate the means; analyzed using Student's *t*‐tests. Abx‐, antibiotics untreated; Abx+, antibiotics treated; GAS, gastrocnemius muscle; SCFA, short‐chain fatty acid; SOL, soleus muscleTA, tibialis anterior muscle; WAT, white adipose tissue.

Antibiotic treatment affects the intestinal microbiome. To explore the role of the microbiome in muscle mass and performance, multiple antibiotics were administered orally to mice (Abx + mice) consuming a standard diet (Figure 1a). After 2 weeks of antibiotic treatment, body weight and WAT mass were comparable between the two groups (Figure 1b,c). Abx + mice had an enlarged cecum, suggesting that undigested dietary fiber was accumulating there owing to disruption of the microbiome (386 ± 106 mg in Abx‐ mice versus 1312 ± 372 mg in Abx + mice, *p* < 0.001; Figure 1e,f). The tibialis anterior muscle was significantly smaller in Abx + mice compared with Abx‐ mice, although no such difference was observed in soleus and gastrocnemius muscles (Figure 1d). Grip strength was significantly lower in Abx + mice compared with Abx‐ mice (108 ± 9 g in Abx‐ mice versus 91 ± 12 g in Abx + mice, *p* < 0.05; Figure 1g). Gut microbes generate SCFAs from fermentable fiber in the intestine. We found that intestinal SCFA content was significantly lower in the Abx + than the untreated group (Figure 1h). Most of the SCFAs produced are used locally in the intestine, but some are absorbed into the circulation. As a result, acetate is present at high concentrations in the plasma (Figure 1i), although plasma acetate concentration was comparable between the two groups (Figure 1i).

### 3.2 Oral supplementation of acetate in antibiotic‐treated mice rescued grip strength

**FIGURE 2:**
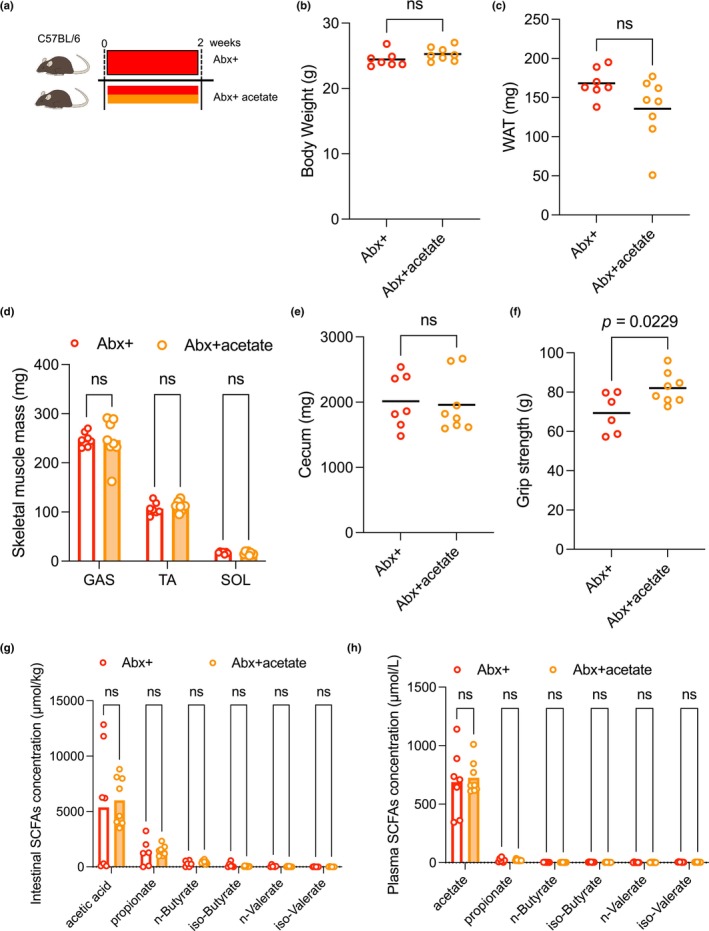
Effects of oral acetate supplementation on antibiotic treatment for 2 weeks. (a) Experimental protocol. C57BL/6 mice were allocated to two groups and administered antibiotics and acetate with antibiotics for 2 weeks (Abx+, *n* = 7; Abx + acetate, *n* = 8). (b) The bodyweight of Abx + and Abx + acetate mice (Abx+, *n* = 7; Abx + acetate, *n* = 8). (c) WAT of Abx + and Abx + acetate mice (Abx+, *n* = 7; Abx + acetate, *n* = 8). (d) Skeletal muscles of Abx + and Abx + acetate mice (Abx+, *n* = 7; Abx + acetate, *n* = 8). (e) Cecum of Abx + and Abx + acetate mice (Abx+, *n* = 7; Abx + acetate, *n* = 8). (f) Grip strength of Abx + and Abx + acetate mice (Abx+, *n* = 6; Abx + acetate, *n* = 8). (*g*) Intestinal SCFA concentrations of Abx + and Abx + acetate mice (Abx+, *n* = 6; Abx + acetate, *n* = 8). (h) Plasma SCFA concentrations of Abx + and Abx + acetate mice (Abx+, *n* = 6; Abx + acetate, *n* = 8). Data expressed as mean; ns, not statistically significant. The lines indicate the means; analyzed using Student's t‐tests. Abx+, antibiotics treated; Abx + acetate, antibiotics with acetate treated; WAT, white adipose tissue; SCFA, short‐chain fatty acid; GAS, gastrocnemius muscle; TA, tibialis anterior muscle; SOL, soleus muscle.

To examine the potential role of intestinal acetate in muscle performance, acetate was supplemented orally to mice (Abx + acetate mice) (Figure 2a). Body weight was not different between the two groups (Figure 2b). Regarding body composition, Abx + acetate mice had similar muscle, WAT, and cecum masses as Abx + mice (Figure 2c–e). Acetate supplementation for 2 weeks with antibiotics significantly prevented the reduction of grip strength observed in Abx + mice (69 ± 10 g in Abx + mice versus 82 ± 14 g in Abx + acetate mice, *p* < 0.05; Figure 2f). We found that intestinal SCFA contents in the Abx + acetate mice were comparable with those in the Abx + group (Figure 2g). Similar to the intestinal content, plasma acetate and other SCFA concentrations were also comparable with those in the Abx + group (Figure 2h).

### 3.3 Antibiotic treatment alters the composition of the microbiome

**FIGURE 3:**
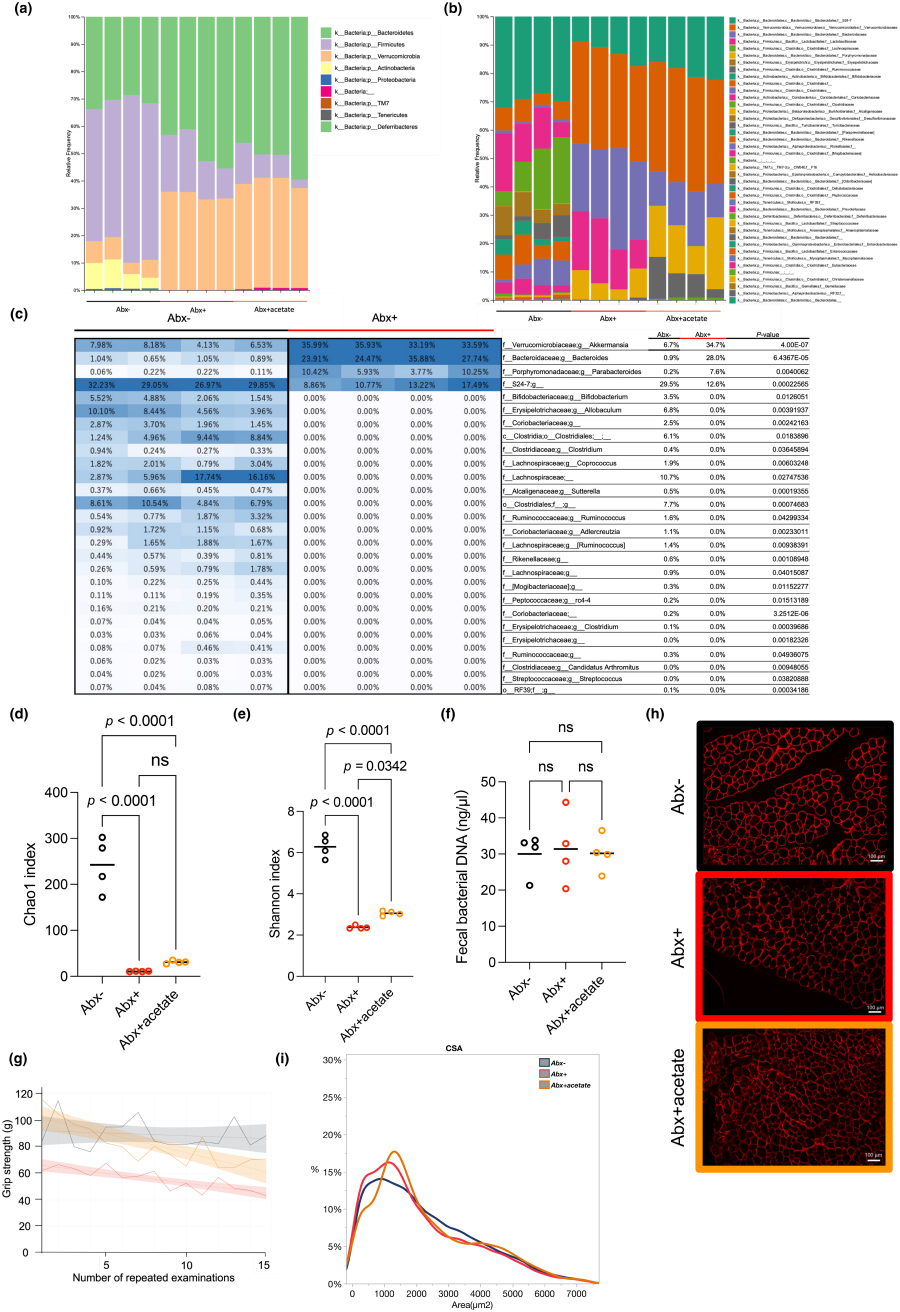
Analysis of the fecal microbiome in Abx‐, Abx+, and Abx + acetate groups. (a) Composition of the fecal microbiome at the phylum level. (b) Composition of the fecal microbiome at the family level. (c) Heat map and list of bacterial taxa showing significant differences in the percentage composition of the entire microbiome, analyzed using the Student's t‐test. Values are averages and *P*‐values. (d and e) Fecal bacterial diversity assessed using the Chao1 and Shannon indices, respectively. (f) Concentration of fecal bacterial DNA. (g) Repeated grip strength test of Abx‐, Abx + and Abx + acetate mice. Spline curves with 95% confidence intervals. (h) Representative cross‐sectional images of the TA from Abx‐, Abx + and Abx + acetate mice. Muscle fibers were immunostained with anti‐laminin antibody in red. *I*: CSA of Abx‐, Abx + and Abx + acetate mice. *n* = 4 per group. Data expressed as mean; ns, not statistically significant; analyzed using one‐way analysis of variance and subsequent post hoc Tukey tests (d and e) or Student's t‐tests (f). Abx + acetate+, antibiotics with acetate treated; Abx‐, antibiotics untreated; Abx+, antibiotics treated; CSA, cross‐sectional area; TA, tibialis anterior muscle.

Next, we determined the effects of antibiotic treatment on the microbiome by analyzing intestinal bacterial composition in the Abx‐, Abx+, and Abx + acetate mice using 16S/rRNA analysis. Compared with Abx‐ mice, Abx + mice had a smaller population of Firmicutes and a larger population of Bacteroidetes than in the control group at phylum level (Figure 3a). However, at family level, Bacteroidales family S24‐7, known as one of the most abundant microbiota in mice (Lagkouvardos et al., 2019), was significantly reduced in cecum contents of Abx + mice compared with untreated mice (Figure 3b). Similarly, at genus level, the acetate‐producing microbiota (Koh et al., 2016) Firmicutes, c_Clostridia, o_Clostridiales, and f_Lachnospiraceae were reduced (Figure 3b). By contrast, Bacteria, p_Bacteroidetes, c_Bacteroidia, o_Bacteroidales, and f_Bacteroidaceae, which are known for producing beta‐lactamase, were increased in the cecum contents of the Abx + mice compared with the Abx‐ mice, suggesting microbial substitution including antimicrobial resistance (Figure 3b). There were 27 microbiota in genus levels that were significantly different between Abx + and Abx‐ mice (Figure 3c). Furthermore, antibiotic treatment significantly reduced fecal bacterial diversity, as assessed using Shannon and Chao indexes (Figure 3d,e), but showed no change in bacterial DNA concentration (Figure 3f). We further evaluated the endurance ability of forearms by the repeated grip strength test and found that oral administration of acetate rescued grip strength initially but reduced grip strength after repetition (Figure 3g). Similarly, the CSA of muscle fibers in the tibialis anterior muscle was smaller in Abx + mice but rescued in the Abx + acetate group (*p* < 0.001; Figure 3h,i). To explorer the effect on muscle fiber type, we examined mRNA expression of myosin heave chains (MYHs) and fond no significant difference between groups (Figure S1: https://doi.org/10.6084/m9.figshare.25665051.v1). We added metagenome analysis using the KEGG pathway, which is related to SCFA metabolism, and found that many enzymes were altered by antibiotic treatment and rescued by oral acetate supplementation (Figure S2; https://exploratory.io/note/hwM3anC2KB/Supplemental‐Figure‐S2‐WEk7Mqa9wF).

### 3.4 Reduction of dietary fiber is associated with lower body weight, WAT mass, and muscle fiber size

**FIGURE 4:**
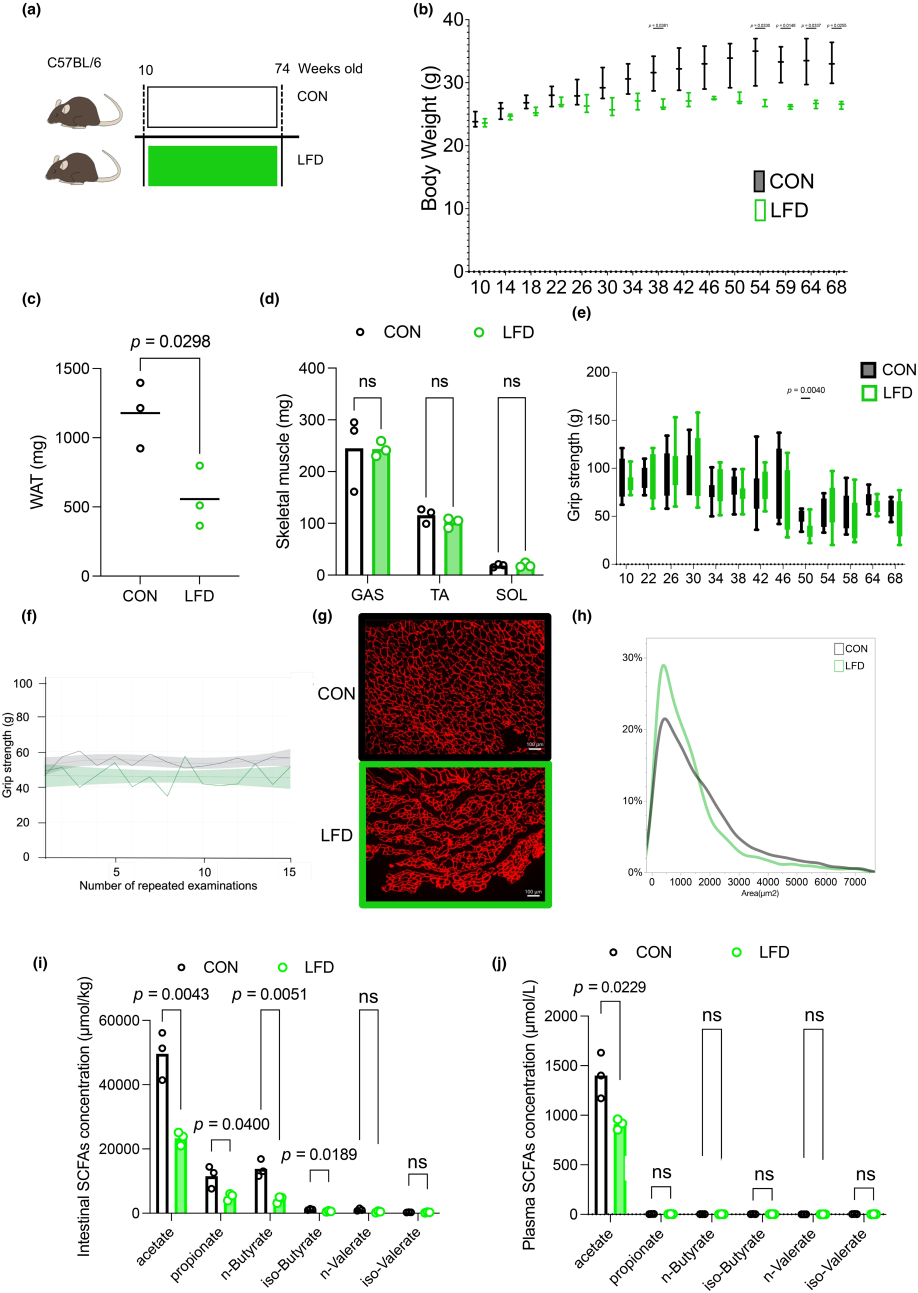
Metabolic phenotype of mice fed an LFD or control diet (CON). (a) Experimental protocol: C57BL/6 mice were allocated to two groups and fed either LFD or CON for 64 weeks starting at 10 weeks of age. *n* = 3 per group. The LFD contained low dietary fiber. (b) The bodyweight of LFD and CON mice. (c) WAT of LFD and CON mice. *D*: Skeletal muscles of LFD and CON mice. (e) Grip strength of LFD and CON mice. *F*: Repeated grip strength test at 58 weeks of age. Spline curves with 95% confidence intervals. *G*: Representative cross‐sectional images of the TA from LFD and CON mice. Muscle fibers are immunostained with anti‐laminin antibody in red. (h) Cross‐sectional area of LFD and CON mice. (i) Intestinal SCFA concentrations of LFD and CON mice. (j) Plasma SCFA concentrations of LFD and CON mice. Data expressed as mean; ns, not statistically significant; analyzed using Student's t‐tests. The boxes indicate the interquartile ranges and the lines within the boxes indicate the medians. Lines show the minimum and maximum values. CON, control diet; CSA, cross‐sectional area; GAS, gastrocnemius muscle; LFD, low‐fiber diet; SCFA, short‐chain fatty acid; SOL, soleus muscle; TA, tibialis anterior muscle; WAT, white adipose tissue.

To explore the relationship between skeletal muscle performance and SCFAs, the long‐term effect of the LFD on skeletal muscle performance was also assessed. Mice were randomly assigned to consume either the control (standard diet) or LFD (1% cellulose) (Figure 4a). Body weight was lower in the LFD group (Figure 4b). Although WAT mass was significantly lower in the LFD group at 74 weeks of age (Figure 4c), skeletal muscle masses were comparable between the two diet groups (Figure 4d). In the control group, grip strength peaked at ~30 weeks of age and gradually reduced by age (Figure 4e). Grip strength in the LFD mice was similar to that in the control mice (Figure 4e) but tended to be lower during the repeated grip strength test (Figure 4f). Muscle fiber was significantly smaller in the LFD group than the control group (Figure 4g,h). We examined mRNA of MYHs and found slight increase in MYH1 but no significant difference in other muscle fiber type (https://doi.org/10.6084/m9.figshare.25665051.v1). Similar to the effect of antibiotic treatment, intestinal SCFA content was significantly lower in the LFD group than the control group (Figure 4i). In addition, plasma acetate concentration was significantly lower in the LFD group (Figure 4j).

### 3.5 Acetyl‐CoA synthase 2 knockdown reduces life span and enhances sarcopenia in mice

**FIGURE 5:**
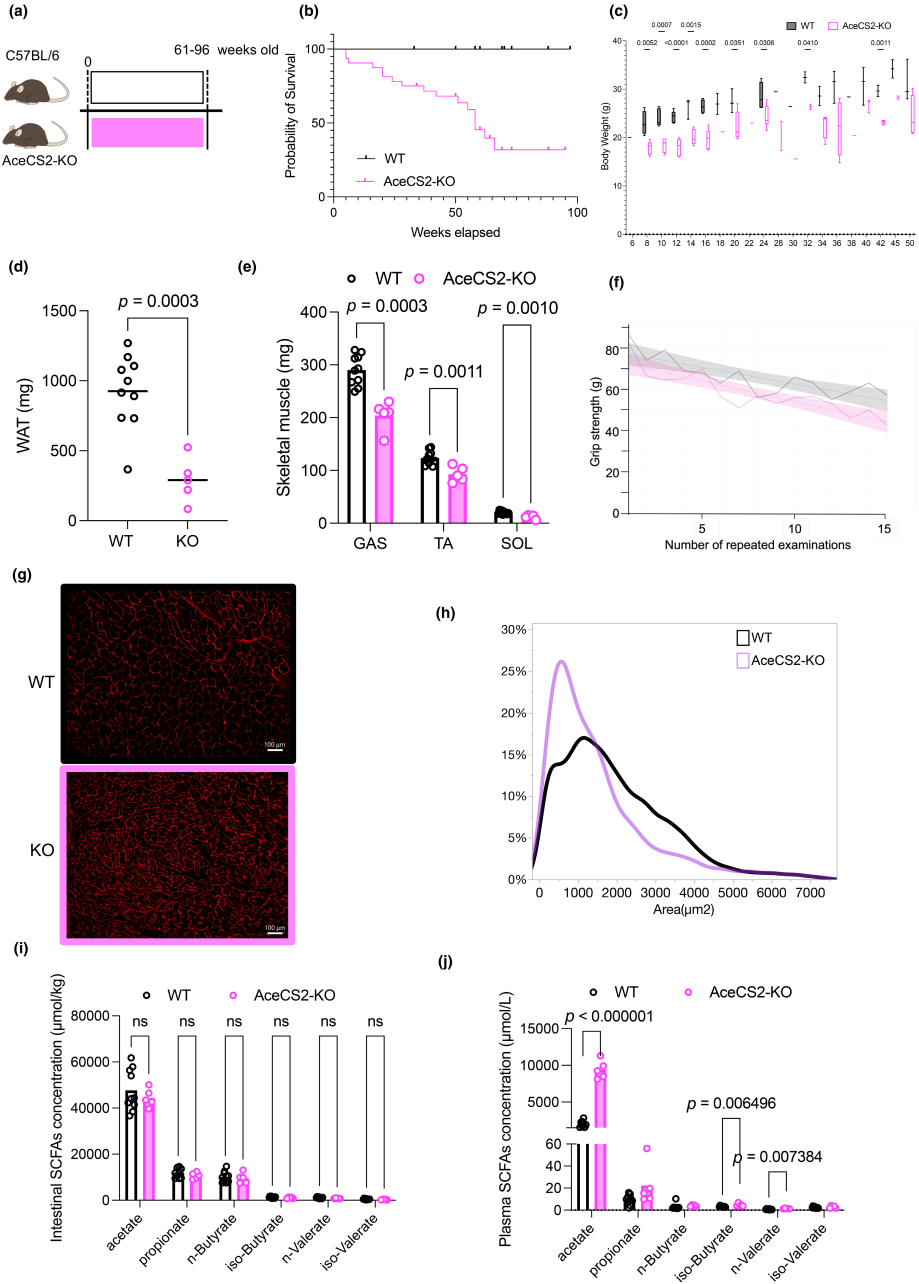
Metabolic phenotype of AceCS2‐KO or WT mice. (a) Mice were fed standard diet for 61–96 weeks. (b) Kaplan–Meier analysis of survival in males of WT and AceCS2‐KO mice (WT, *n* = 22; AceCS2‐KO, *n* = 32). (c) The bodyweight of WT and AceCS2‐KO mice (WT, *n* = 10; AceCS2‐KO, *n* = 14). (d) WAT mass of WT and AceCS2‐KO mice (WT, *n* = 10; AceCS2‐KO, *n* = 5). (e) Skeletal muscle masses of WT and AceCS2‐KO mice (WT, *n* = 10; AceCS2‐KO, *n* = 5). (f) Repeated grip strength test. Spline curves with 95% confidence intervals. (g) Representative cross‐sectional images of the TA from WT and AceCS2‐KO mice. Muscle fibers are immunostained with anti‐laminin antibody in red. (h) Cross‐sectional area of WT and AceCS2‐KO mice. (i) Intestinal SCFA concentrations of WT and AceCS2‐KO mice (WT, *n* = 10; AceCS2‐KO, *n* = 5). (j) Plasma SCFA concentrations of WT and AceCS2‐KO mice (WT, *n* = 10; AceCS2‐KO, *n* = 5). Data expressed as mean; ns, not statistically significant; analyzed using Student's *t*‐test (c, d, e, i and j). Lines show the minimum and maximum values. AceCS2, acetyl‐CoA synthetase 2; CSA, cross‐sectional area; GAS, gastrocnemius muscle; SCFA, short‐chain fatty acid; SOL, soleus muscle; TA, tibialis anterior muscle; WAT, white adipose tissue; WT, wild type.

To explore the impact of acetate in skeletal muscle as an energy source, we analyzed acetyl‐CoA synthase 2 knockout (AceCS2‐KO) mice until they were 100 weeks old (Figure 5a). Surprisingly, AceCS2‐KO mice had a shorter life than their wild type littermates (WT) (Figure 5b). Body weight was lower in AceCS2‐KO mice than WT mice (Figure 5c). Surviving mice were evaluated for skeletal muscle performance and sacrificed for further analysis. WAT and skeletal muscle masses were lower in AceCS2‐KO compared with WT mice at 72 weeks of age (Figure 5d,e). Grip strength in the AceCS2‐KO mice was similar to that in the WT mice (78.5 ± 4.8 g in WT mice versus 74.1 ± 4.4 g in AceCS2‐KO mice) but the repeated grip strength test showed slightly faster fatigue in AceCS2‐KO than WT mice (Figure 5f). Muscle fiber area was significantly smaller in AceCS2‐KO compared with WT mice (Figure 5g,h). Muscle fiber type was evaluated by mRNA expression of MYHs and MYH4 was significantly lowered in AceCS2‐KO mice compare do WT suggesting lower Type IIB fiber in AceCS2‐KO (https://doi.org/10.6084/m9.figshare.25665051.v1). AceCS2‐KO and WT mice had comparable intestinal and plasma concentrations of SCFAs, except for plasma acetate concentrations that were higher in the AceCS2‐KO mice because of blunted acetate utilization (Figure 5i,j).

### 3.6 No evidence of enhanced proteasomal catabolic genes in the skeletal muscle of antibiotic‐treated, acetate‐supplemented, or AceCS2‐KO mice

**FIGURE 6:**
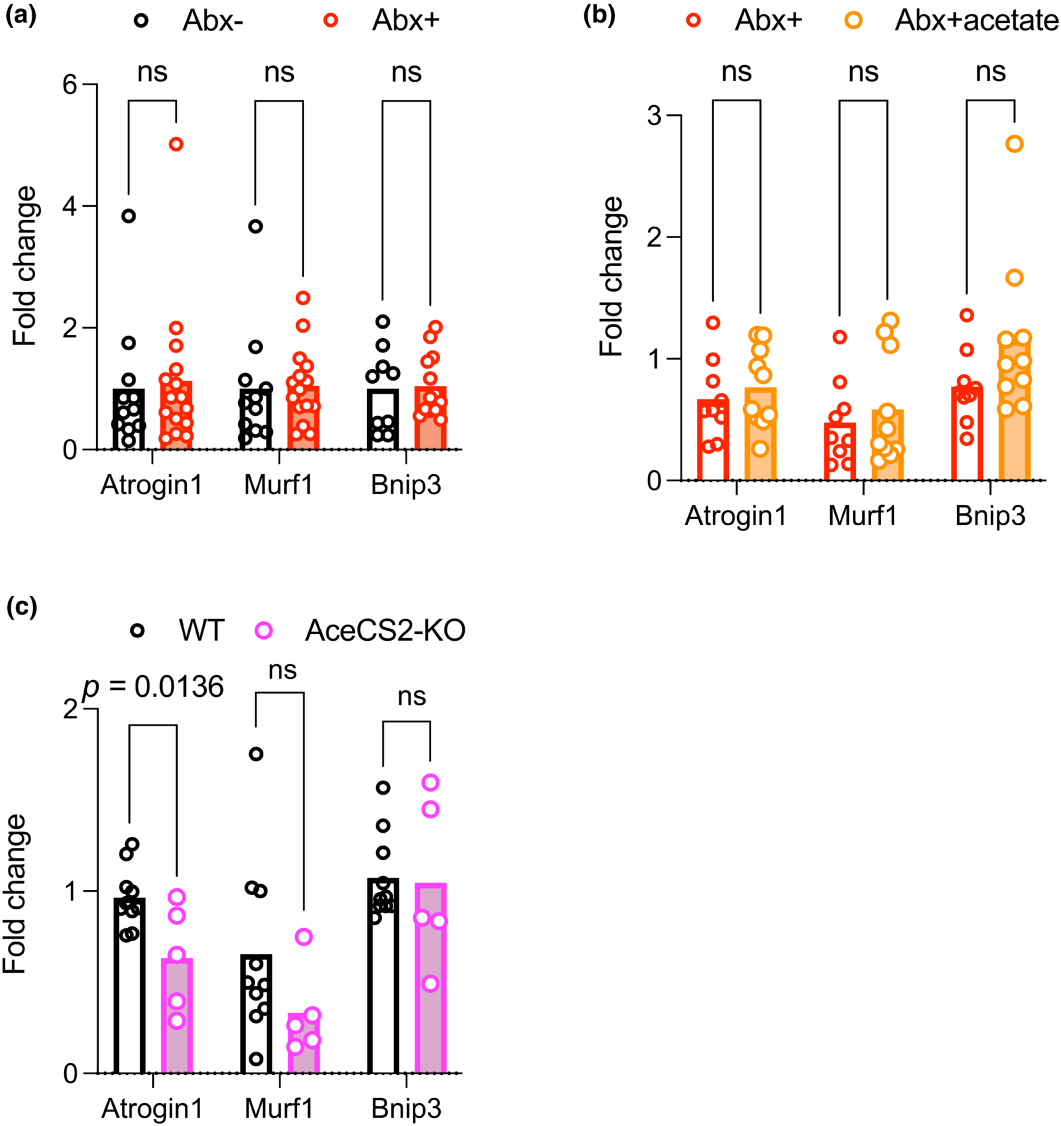
Expression profile of genes related to skeletal muscle degradation in TA muscle. Quantitative PCR analysis of intracellular ubiquitin ligases‐muscle atrophy F‐box (Atrogin1/MAFbx), muscle RING finger 1 (Murf1), and the autophagy‐related gene, Bcl‐2 and 19‐kDa interacting protein 3 (Bnip3). (a) Abx‐ (*n* = 10) and Abx + mice (*n* = 15). (b) Abx + (*n* = 8), Abx + acetate (*n* = 10). (c) WT (*n* = 7), AceCS2‐KO (*n* = 5). Data expressed as mean; ns, not statistically significant. Analyzed using Student's *t*‐test. TA muscles were harvested after 12 h of fasting (*a* and *b*) and in ad libitum conditions (*c*). TA, tibialis anterior muscle.

To investigate the molecular mechanisms of how SCFAs regulate skeletal muscle fibers, we examined proteasomal and autophagy pathways by measuring mRNA expression of intracellular ubiquitin ligases‐muscle atrophy F‐box (Atrogin1/MAFbx), muscle RING finger 1 (Murf1), and Bcl‐2 and 19‐kDa interacting protein 3 (Bnip3). These genes are stimulated by prolonged fasting as previously reported (Figure S3: https://doi.org/10.6084/m9.figshare.25674135). Abx + mice showed no significant difference from the untreated group (Figure 6a). Similarly, Abx + acetate mice showed similar expression levels compared with Abx + mice (Figure 6b). AceCS2‐KO mice had lower Atrogin1/ MAFbx expression levels but similar Murf 1 and Bnip 3 expression levels compared with WT mice (Figure 6c).

## 4 DISCUSSION

There were two important findings of this study in mice. First, acetate from the intestine had a role in maintaining skeletal muscle performance. Second, plasma acetate maintained skeletal muscle performance as an energy source in skeletal muscle.

In this study, acetate from the intestine had a role in maintaining skeletal muscle performance measured by grip strength (Figures 1g, 2f, and 3g) and muscle fiber area (Figures 3i, 4h, and 5h). These findings are consistent with those of previous studies using germ‐free mice (Lahiri et al., 2019), other dietary interventions (Lahiri et al., 2019), or antibiotic treatment (Nay et al., 2019). Previously our group also reported that antibiotic treatment in mice reduced running time on a treadmill and that this phenomenon was rescued by an intra‐subcutaneous infusion of acetate or fecal transplantation from control mice (Okamoto et al., 2019). In humans, an association has been observed between intake of SCFAs and muscle strength loss among community‐dwelling Japanese adults (Otsuka et al., 2023).

Plasma acetate is an important energy source in skeletal muscle under physiological condition, at least during endurance exercise. Our previous study demonstrated that acetate infusion normalizes endurance exercise performance, which is impaired by antibiotic treatment (Okamoto et al., 2019). This result is consistent with that of a previous study in which AceCS2‐KO mice had low exercise tolerance in a fasting state, as assessed using a similar protocol (Sakakibara et al., 2009). AceCS2 is the only enzyme that can convert acetate into acetyl‐CoA in mitochondria (Fujino et al., 2001), while the liver can generate acetate during starvation from the beta‐oxidation of fatty acids, using AceCS1 (Wang et al., 2023). In mice, acetate turnover has been estimated to be ~20 nmol/g/min by the infusion of isotope‐labeled acetate, suggesting very rapid turnover (Perry et al., 2016). In humans, 13C acetate is commonly used to measure gastric emptying because of its fast absorption and oxidation from the upper intestine (Braden et al., 1995). We did not observe an increase in plasma and cecum acetate concentrations in the Abx + acetate group, probably due to a faster turnover (Figure 2h). In horses, acetate infusion during exercise reduces plasma‐free fatty acid and glycerol concentrations, which may be because of a reduction in lipolysis (Pratt et al., 2005), and oral administration of acetate rapidly restores muscle glycogen (Waller et al., 2009). In humans, acetate infusion increases acetyl‐CoA concentrations in skeletal muscle (Evans et al., 2001). These findings indicate that plasma acetate is an energy source for skeletal muscle.

Microbiota was broadly affected by the antibiotic treatment (Figure 3a,b). At family level, multiple acetate‐generating microbiota such as S24‐7 (Miyamoto et al., 2023), Bifidobacterium, Clostridium, Ruminococcus, and Lactobacillales Streptococcus (Koh et al., 2016) were attenuated by antibiotic treatment, which was consistent with a reduced cecum acetate concentration (Figure 3c). However, some major acetate‐generating microbiota such as Bacteroidetes and Akkermansia were increased by antibiotic treatment (Figure 3c). Similarly, propionate‐generating microbiota such as Ruminococcus (Koh et al., 2016) were also attenuated (Figure 3c). Butyrate is either generated through a butyrate kinase route or the butyl‐Coa:acetate CoA‐transferase route. Coprococcus, which generates butylate from acetate (Koh et al., 2016), was also attenuated by antibiotics (Figure 3c). By contrast, Akkermansia, Bacteroides, Parabacteroides, S24‐7, and Ruminococus were rescued by oral acetate administration, suggesting that oral acetate affects microflora and its effect on skeletal muscle performance. Metagenome analysis using the KEGG pathway showed that multiple enzymes related to SCFA metabolism were altered by antibiotic treatment and rescued by oral acetate supplementation (Figure S2; https://exploratory.io/note/hwM3anC2KB/Supplemental‐Figure‐S2‐WEk7Mqa9wF). Overall, antibiotic treatment affected a broad range of microbiota and reduced microbiota diversity in mice, subsequently attenuating intestinal concentrations of SCFAs leading to reduce availability to skeletal muscle.

AceCS2‐KO mice had shorter lifespans in our study (Figure 5a,b). We have no direct evidence why AceCS2‐KO mice died earlier than their WT littermates, but the mice that survived were significantly leaner than WT mice, mainly because of reduced WAT mass. There was significant difference in skeletal muscle mass, and muscle fiber area was significantly smaller in AceCS2‐KO than WT mice (Figure 5g,h). AceCS‐2 is the only enzyme that uses acetate in the TCA cycle as a direct energy source; therefore, extremely high concentrations of plasma acetate were observed in AceCS2‐KO mice (Figure 5j). In addition to the skeletal muscle, decreased in WAT mass were observed among Abx, LFD, and AceCS2‐KO mice models. Direct effect of acetate in adipose tissues may also contribute to reduced adiposity in these models. Acetylation is a known epigenetic modulator (Kaelin William & McKnight, 2013). Thus, it is also possible that high acetate but low acetyl‐CoA concentrations (Sakakibara et al., 2009) might affect the epigenetic landscape and thereby reduce lifespan in these mice.

Body weight is an important factor for muscle mass and grip strength. Treatment of young mice with antibiotics for 2 weeks resulted in a significant decrease in TA and GAS muscle mass and grip strength normalized for body weight (Figure S4A–D: https://doi.org/10.6084/m9.figshare.25672272.v1). Experiments on low‐fiber aged mice showed no significant differences in skeletal muscle mass and grip strength when normalized for body weight (Figure S4E,F: https://doi.org/10.6084/m9.figshare.25672272.v1). In aged AceCS2‐KO mice, there was no difference in muscle mass after normalization for body weight, but grip strength was higher (Figure S4G,H: https://doi.org/10.6084/m9.figshare.25672272.v1). Taken together, the possibility remains that our experimental results were mediated by changes in body weight.

There are several limitations to this study. First, the plasma acetate concentration was merely changed by antibiotics, oral acetate administration, low fiber diet. A time course experiment of oral acetate administration reported no increase in chronic administration but ~60 minutes increase with a bolus oral or intraperitoneal treatment (Shubitowski et al., 2019), supporting acetate has very rapid turnover rate (Perry et al., 2016). Second, the low‐fiber diet we chose has less dietary fiber than the regular diet, but also has less protein content. Differences in protein content may affect muscle performance. Third, there were discrepancies between experimental conditions for muscle mass, CSA of muscle fiber, grip strength, and grip strength repetition test. Since muscle mass includes not only muscle fibers but also adipose tissue surrounding the muscle, which is particularly typical of aged muscle (Nilwik et al., 2013), we believe that the CSA of muscle fibers is more closely related to grip strength. Duration of treatment, age, and strain may also influence this discrepancy. It is suggested that macronutrients govern muscle performance, but that SCFA may additionally influence muscle performance under some circumstances, such as aging or antibiotic administration. Fourth, the molecular mechanism by which acetate maintains muscle performance remains unknown. Acetate is a good energy substrate in the brain and stimulates motivation of exercise during endurance training (Agirman & Hsiao, 2022) or appetite in mice (Perry et al., 2016). Thus, it is possible that reduced grip strength and endurance in repeated grip strength tests may not be via skeletal muscle but via the brain. We speculate that acetate acts directly as an energy substrate in skeletal muscle, however, this is one of the limitations of this study, as we have not directly measured skeletal muscle force or torque in ex vivo. At least, ubiquitin – proteasome/autophagy genes were not a mechanistic factor in our experimental setting (Figure 6). GPR41/43 signaling also may not be a mechanistic factor because extremely high acetate concentration did not increase CSA in AceCS2 knockout mice (Figure 5h). Insulin/insulin like growth factor‐1/mechanistic target of rapamycin may be a remaining pathway that increases muscle mass at the postprandial stage (Liu et al., 2021). Further experiments are necessary to clarify this possibility.

From a clinical point of view, antibiotics are often used to treat infectious diseases and in the postoperative stage in patients but are a known risk factor for sarcopenia (Welch et al., 2018). Inpatients are often immobilized or experiencing undernutrition; therefore, dysbiosis and reduced SCFA may have an impact on muscle weakness in these patients. Chronic inflammation is considered a molecular mechanism that underlines weakness in hospitalized individuals (Bano et al., 2017), but we propose a direct effect of SCFA on skeletal muscles as an alternative mechanism. Microbiota and acetate may be a potential new therapeutic target for sarcopenia and dynapenia in humans.

In this mouse study, oral antibiotics reduced grip strength, muscle fiber area, and SCFA concentration in cecum. Furthermore, experiments with oral acetate supplementation in antibiotic‐treated mice, prolonged LFD treatment, and AceS2‐KO suggested the importance of acetate on the skeletal muscle. In conclusion, we reveal a key role of acetate derived from the intestine in maintaining skeletal muscle performance in mice.

## AUTHOR CONTRIBUTIONS

S.K. performed the experiments, designed the project, and wrote and edited the manuscript. K. Morino interpreted the results, designed the project, and wrote and edited the manuscript. T.O. interpreted the results. T.M. performed the experiments. S.I., N.O., K. Murata, T.Y., J.S., and Y.F. discussed the results and commented on the manuscript. S.K. and H.M. reviewed the manuscript and directed all the work.

## FUNDING INFORMATION

This study was funded by the Shiga University of Medical Science.

## CONFLICT OF INTEREST STATEMENT

No conflicts of interest, financial or otherwise, are declared by the authors.

## ETHICS STATEMENT

Animal handling and experimentation were conducted in accordance with the guidelines of the Research Center for Animal Life Science at Shiga University of Medical Science (Approved number #29‐23, #3‐41). All experimental protocols were approved by the Gene Recombination Experiment Safety Committee and the Research Center for Animal Life Science at Shiga University of Medical Science (Approved number #2020‐4‐4, 2022‐7‐4, 2023‐6‐1).

## Supporting information


**Table S1:** https://doi.org/10.6084/m9.figshare.25672320.v1. Nutritional information for the mouse diets.

**Table S2:** https://doi.org/10.6084/m9.figshare.25672338.v1. Primer sequences.

**Figure S1:** https://doi.org/10.6084/m9.figshare.25665051.v1. Expression profile of genes related to skeletal muscle fiber type.

**Figure S2:** https://exploratory.io/note/hwM3anC2KB/Supplemental‐Figure‐S2‐WEk7Mqa9wF. The metagenome analysis using the Kyoto Encyclopedia of Genes and Genomes pathway related to short‐chain fatty acid metabolism.

**Figure S3:** https://doi.org/10.6084/m9.figshare.25674135. Expression of proteasomal catabolic genes in the skeletal muscle during prolonged fasting.

**FIGURE S4:** https://doi.org/10.6084/m9.figshare.25672272.v1. Skeletal muscle masses and Grip strength per bodyweight.

## Data Availability

Representative underlying imaging data sets generated and analyzed during the current study are available in the Supplemental Material: https://figshare.com/s/aa961d7f71e6191260f9
