# Correction to “Acetate derived from the intestinal tract has a critical role in maintaining skeletal muscle mass and strength in mice”

**DOI:** 10.14814/phy2.16135

**Published:** 2024-07-02

Saki Kobayashi, Katsutaro Morino, Takuya Okamoto, Mitsumi Tanaka, Shogo Ida, Natsuko Ohashi, Koichiro Murata, Tsuyoshi Yanagimachi, Juro Sakai, Hiroshi Maegawa, Yukihiro Fujita, Shinji Kume. Physiol Rep. 2024 Jun;12(11):e16047. doi: 10.14814/phy2.16047.

In paragraph 3 of the “Materials and Methods” section, the text “the guidelines of the Research Center for Animal Life Science at Shiga University of Medical Science (Approved number #29‐23, #3‐41).” and “the Gene Recombination Experiment Safety Committee and the Research Center for Animal Life Science at Shiga University of Medical Science (Approved number #2020‐4‐4, 2022‐7‐4, 2023‐6‐1)” was incorrect. This should have read: “the guidelines of the Research Center for Animal Life Science at Shiga University of Medical Science (Approved number #2020‐4‐4, 2022‐7‐4, 2023‐6‐1).” and “the Gene Recombination Experiment Safety Committee and the Research Center for Animal Life Science at Shiga University of Medical Science (Approved number #29‐23, #3‐41).”

We apologize for this error.

