# Supplementary figures and images for: Acetate derived from the intestinal tract has a critical role in maintaining skeletal muscle mass and strength in mice
Source: Physiol Rep. 2024 Jun 4;12(11):e16047. doi: 10.14814/phy2.16047 (PMC11150057; doi:10.14814/phy2.16047)

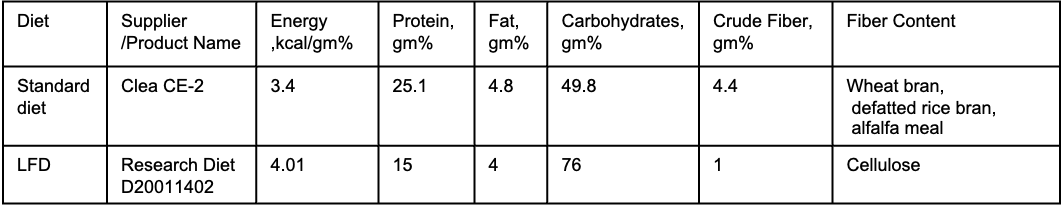


Supplemental Table S1. Nutritional information for the mouse diets

Supplement: Supplementary file 1 — Table S1: https://doi.org/10.6084/m9.figshare.25672320.v1. Nutritional information for the mouse diets. [file PHY2-12-e16047-s006.docx]

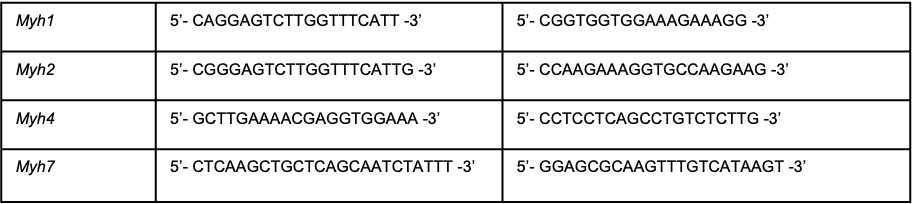

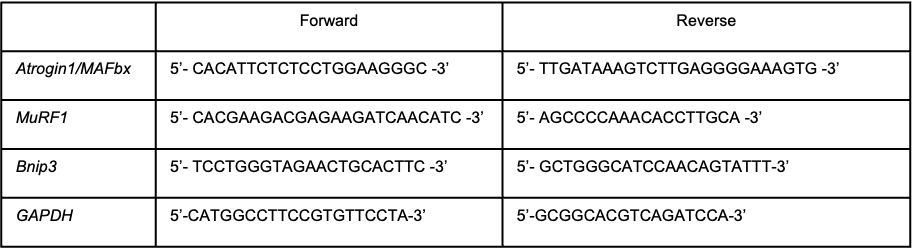


Supplemental Table S2. Primer sequences

Supplement: Supplementary file 2 — Table S2: https://doi.org/10.6084/m9.figshare.25672338.v1. Primer sequences. [file PHY2-12-e16047-s004.docx]
