# Supplementary material for: Acetate derived from the intestinal tract has a critical role in maintaining skeletal muscle mass and strength in mice
Source: Physiol Rep. 2024 Jun 4;12(11):e16047. doi: 10.14814/phy2.16047 (PMC11150057; doi:10.14814/phy2.16047)
Supplement: Supplementary file 3 — Figure S1: https://doi.org/10.6084/m9.figshare.25665051.v1. Expression profile of genes related to skeletal muscle fiber type. [file PHY2-12-e16047-s002.pdf]

Supplemental Figure S1

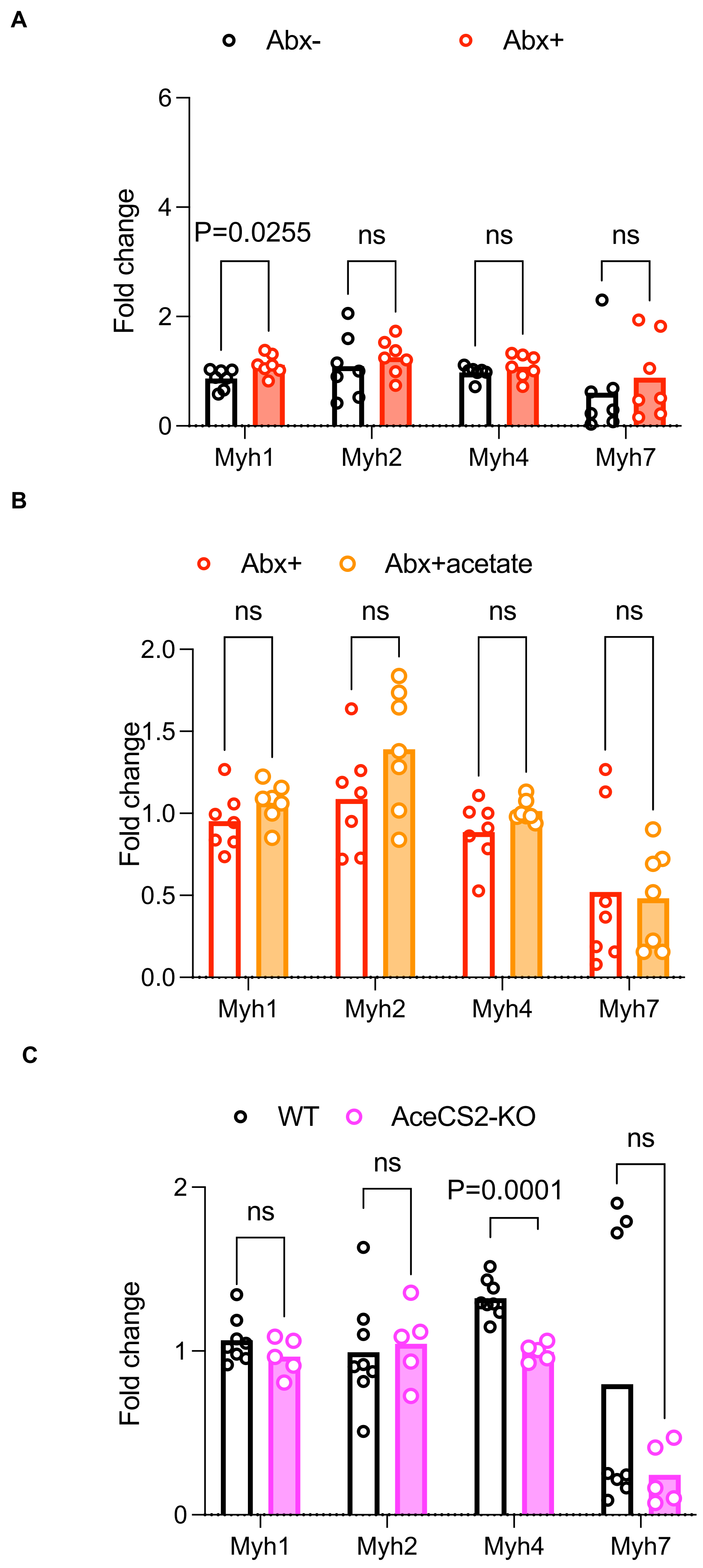

Supplemental Figure S1. Expression profile of genes related to skeletal muscle fiber type in TA muscle. Quantitative PCR analysis of Myh1, Myh2, Myh4, and Myh7. *A*: Abx- (n=7) and Abx+ mice (n=7). *B*: Abx+ (n=7), Abx+ acetate (n=7). *C*: WT (n=8), AceCS2-KO (n=5). Data expressed as mean; ns, not statistically significant. Analyzed using Student's t-test. TA muscles were harvested after 12 hours of fasting (*A* and *B*) and in ad libitum conditions (*C*). TA: tibialis anterior muscle.
