# Supplementary material for: Acetate derived from the intestinal tract has a critical role in maintaining skeletal muscle mass and strength in mice
Source: Physiol Rep. 2024 Jun 4;12(11):e16047. doi: 10.14814/phy2.16047 (PMC11150057; doi:10.14814/phy2.16047)
Supplement: Supplementary file 4 — Figure S2: https://exploratory.io/note/hwM3anC2KB/Supplemental‐Figure‐S2‐WEk7Mqa9wF. The metagenome analysis using the Kyoto Encyclopedia of Genes and Genomes pathway related to short‐chain fatty acid metabolism. [file PHY2-12-e16047-s005.pdf]

Supplement Figure S2

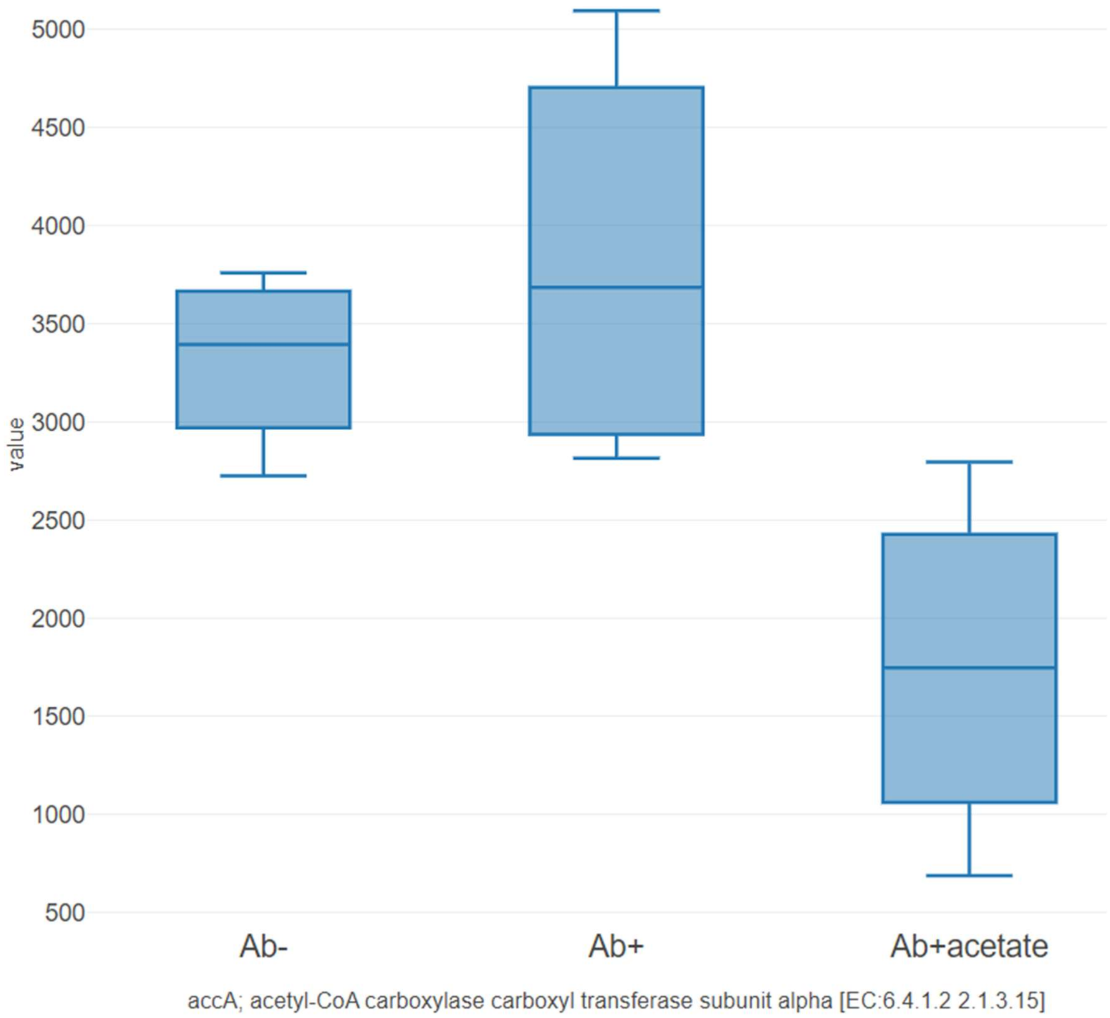

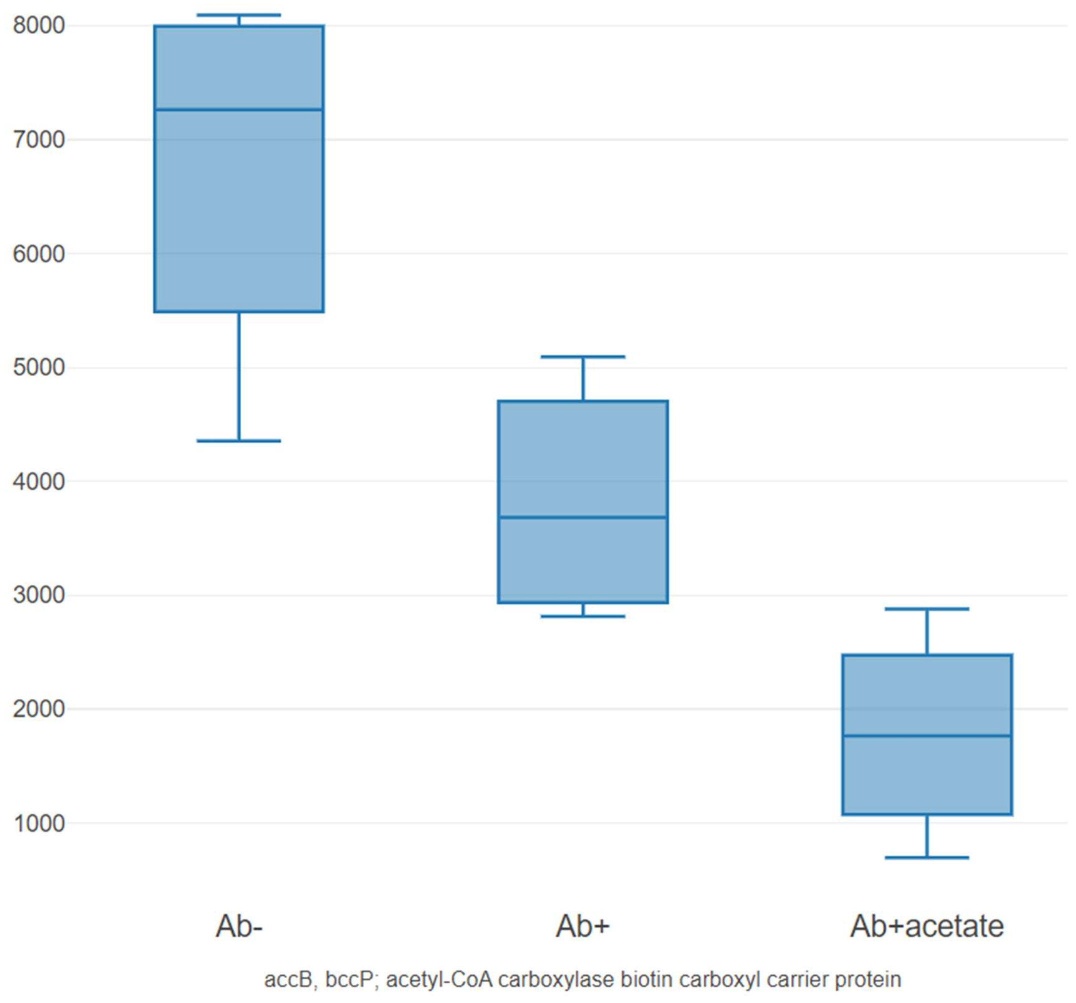
8000
7000
6000
5000
4000
3000
2000
1000
Ab-
Ab+
Ab+acetate
accB, bccP; acetyl-CoA carboxylase biotin carboxyl carrier protein

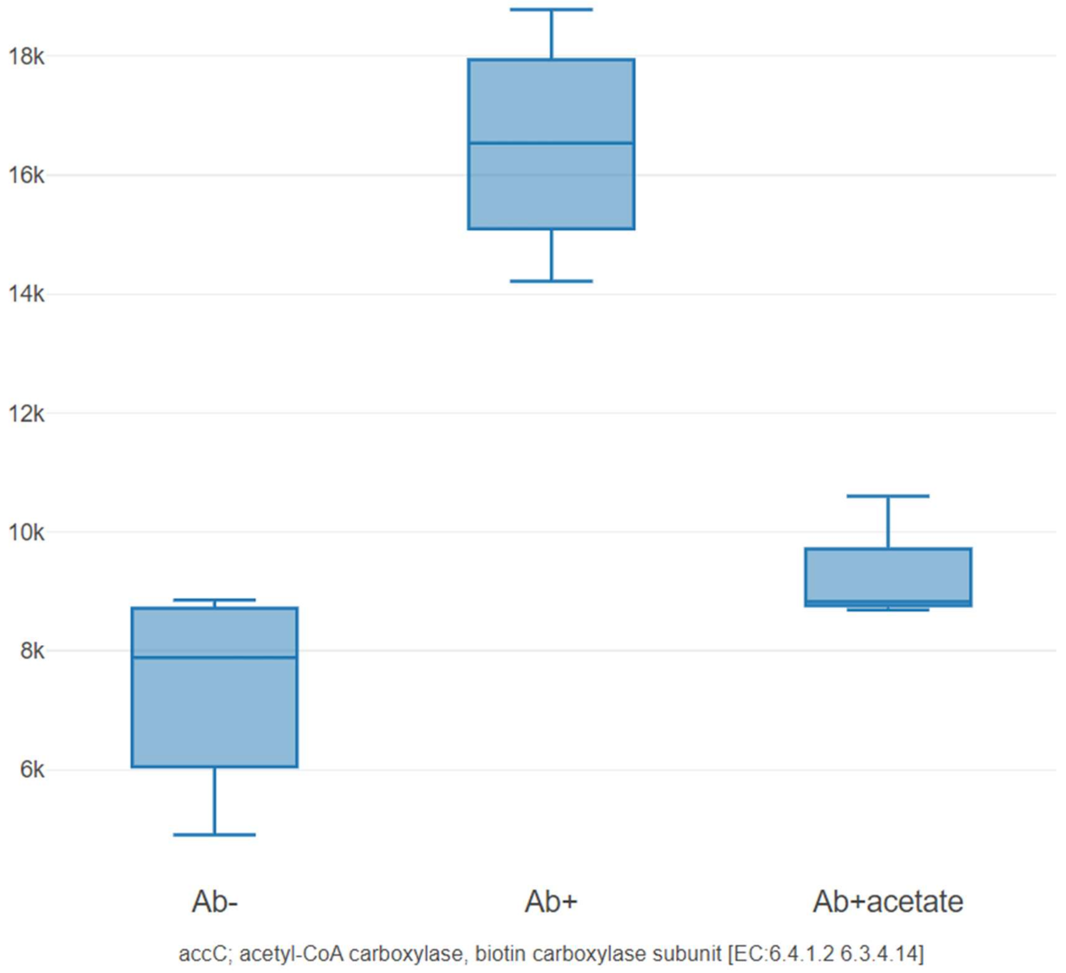
18k
16k
14k
12k
10k
8k
6k
Ab-
Ab+
Ab+acetate
accC; acetyl-CoA carboxylase, biotin carboxylase subunit [EC:6.4.1.2 6.3.4.14]

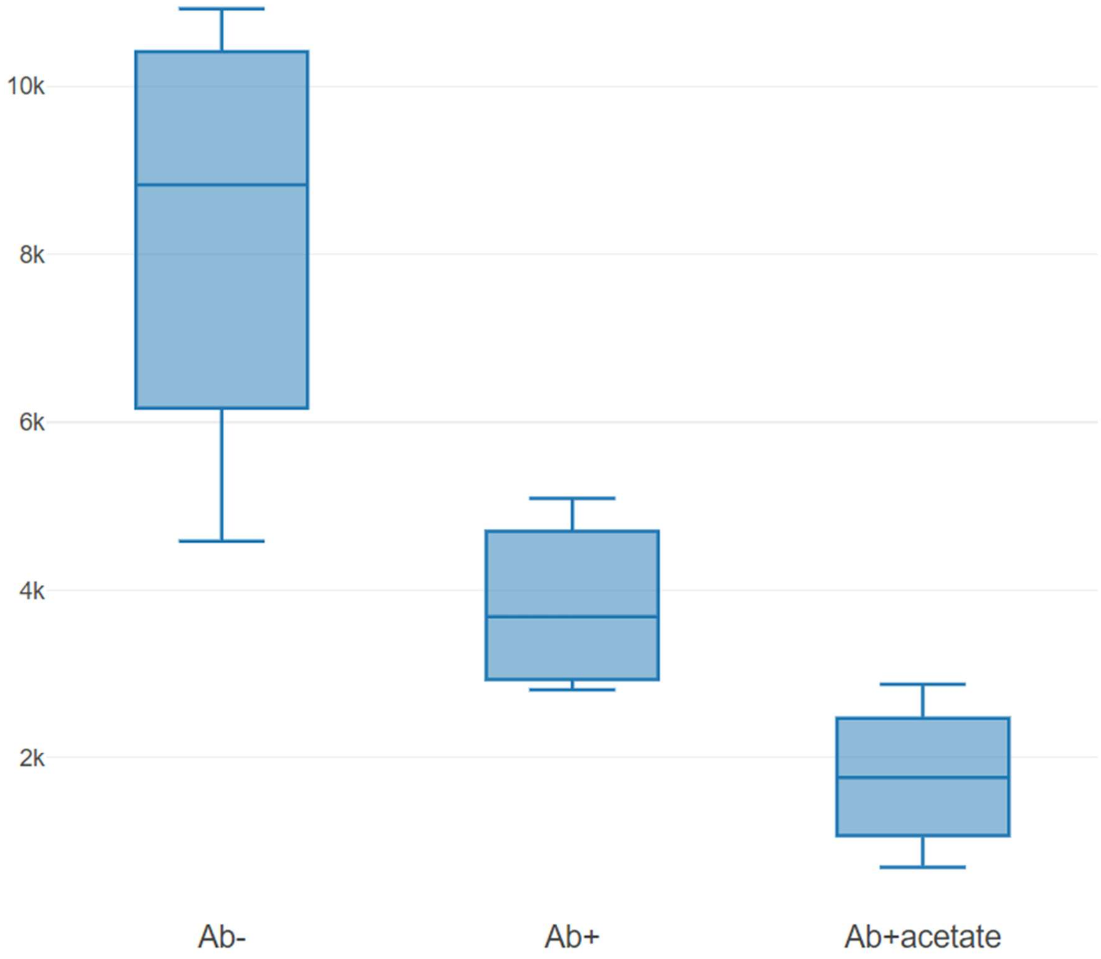

accD; acetyl-CoA carboxylase carboxyl transferase subunit beta [EC:6.4.1.2 2.1.3.15]

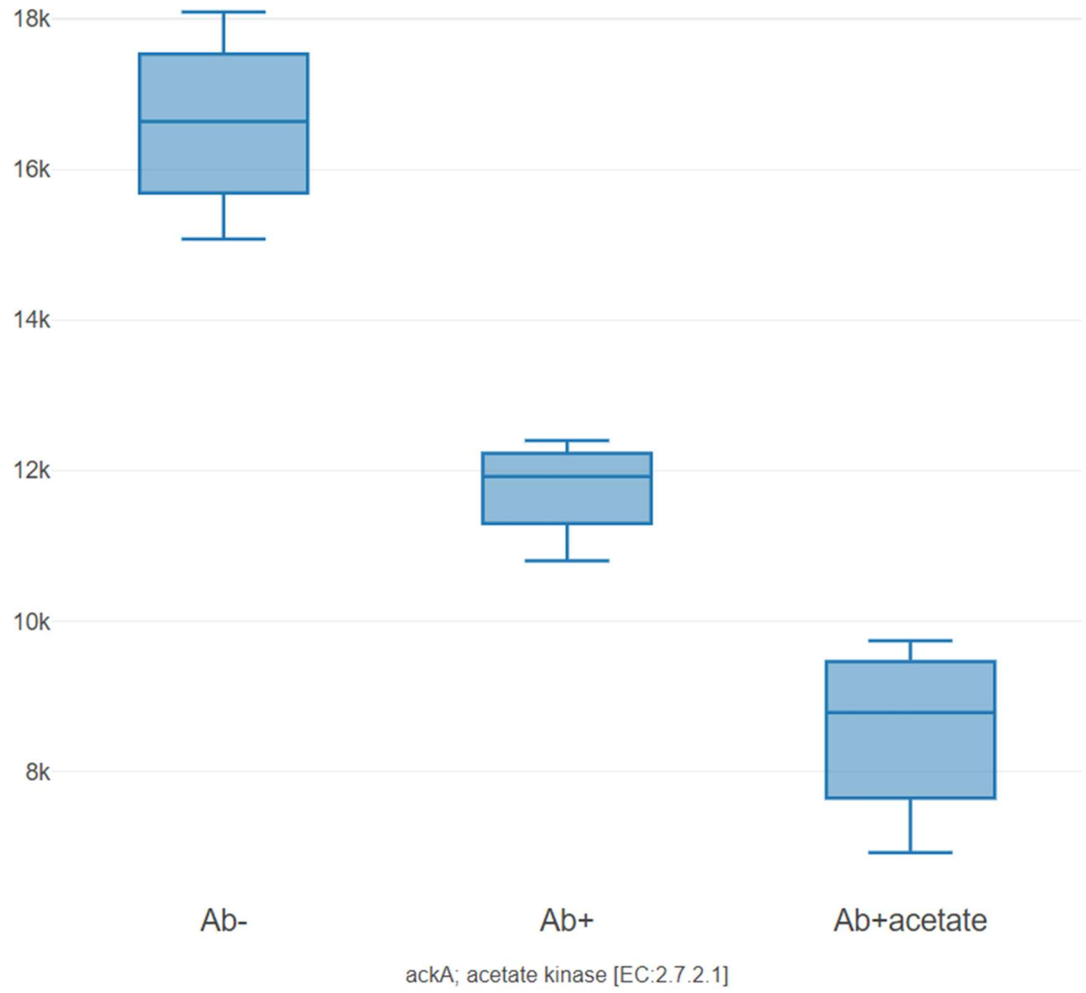
18k
16k
14k
12k
10k
8k
Ab-
Ab+
Ab+acetate
ackA; acetate kinase [EC:2.7.2.1]

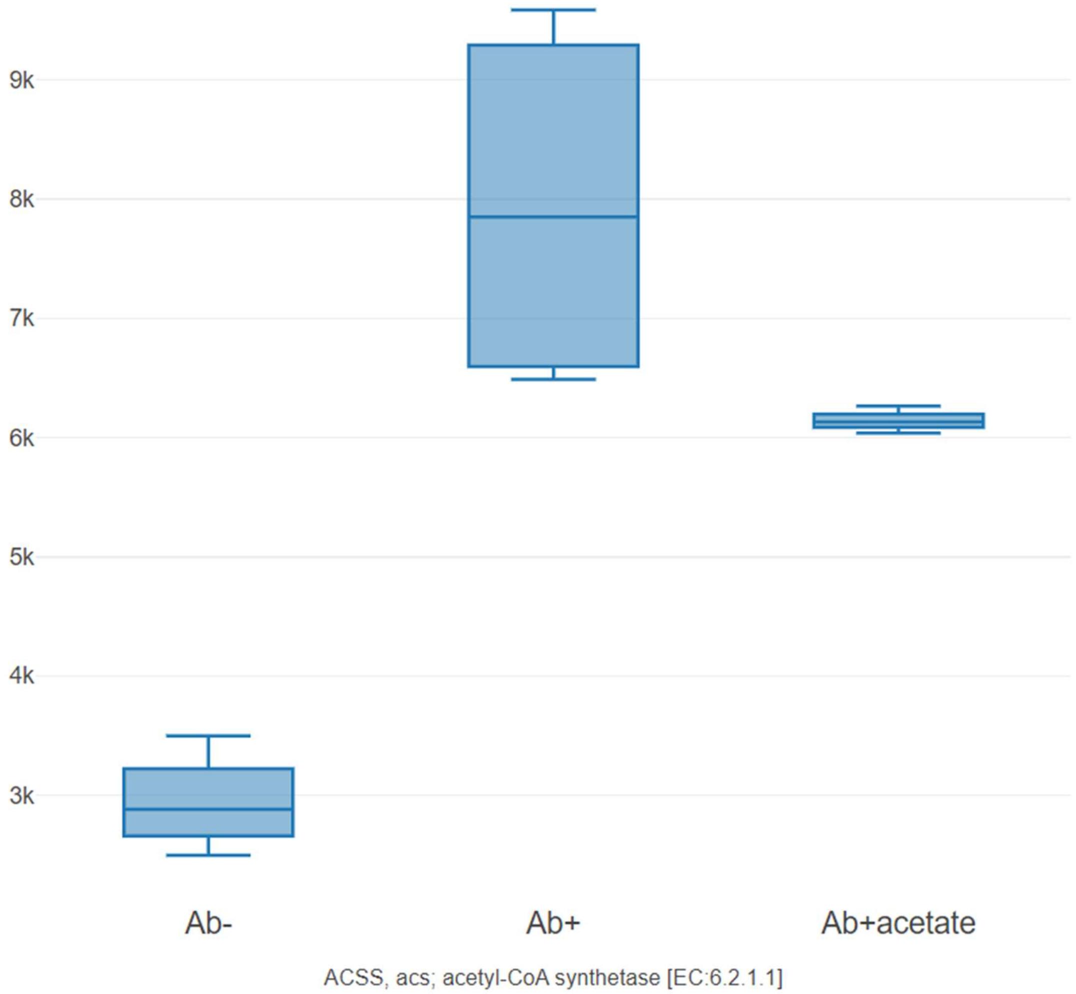
9k
8k
7k
6k
5k
4k
3k
Ab-
Ab+
Ab+acetate
ACSS, acs; acetyl-CoA synthetase [EC:6.2.1.1]

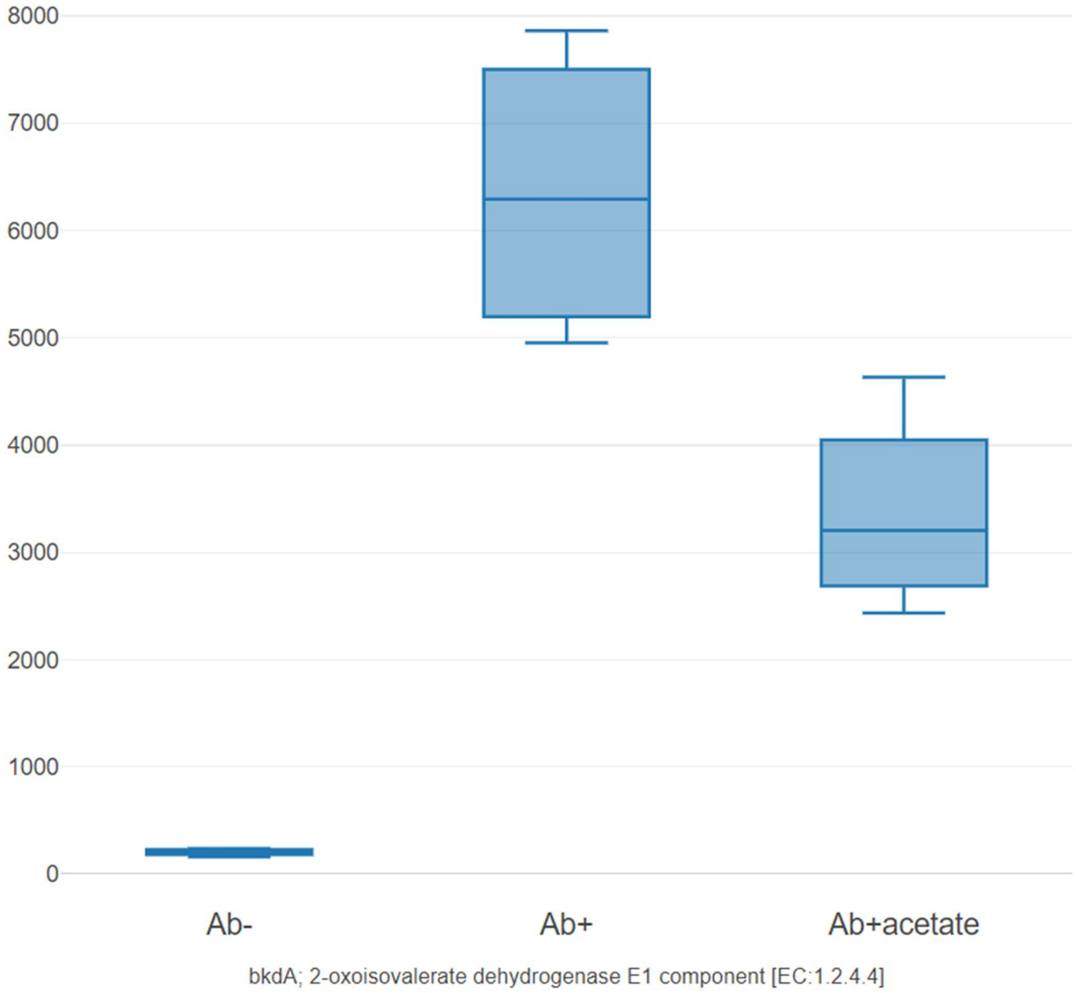
8000
7000
6000
5000
4000
3000
2000
1000
0
Ab-
Ab+
Ab+acetate
bkdA; 2-oxoisovalerate dehydrogenase E1 component [EC:1.2.4.4]

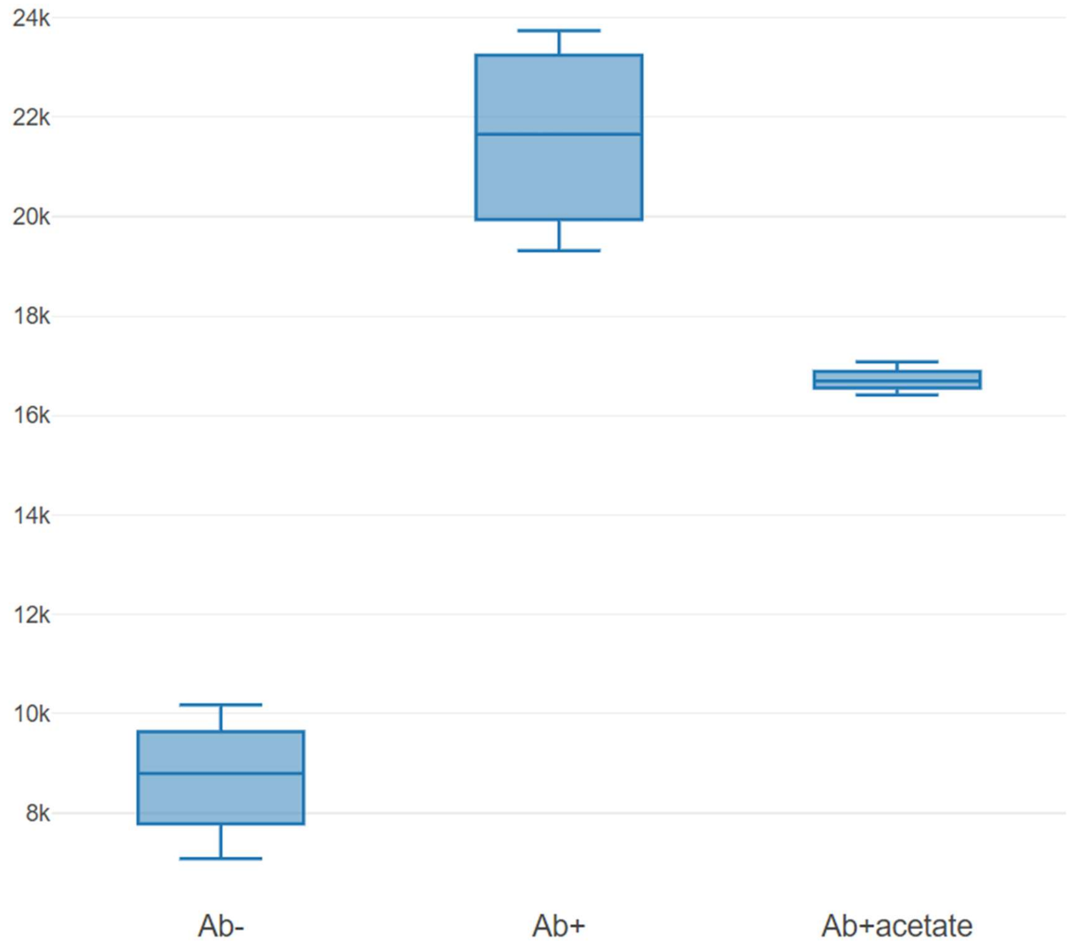

DLD, lpd, pdhD; dihydrolipoamide dehydrogenase [EC:1.8.1.4]

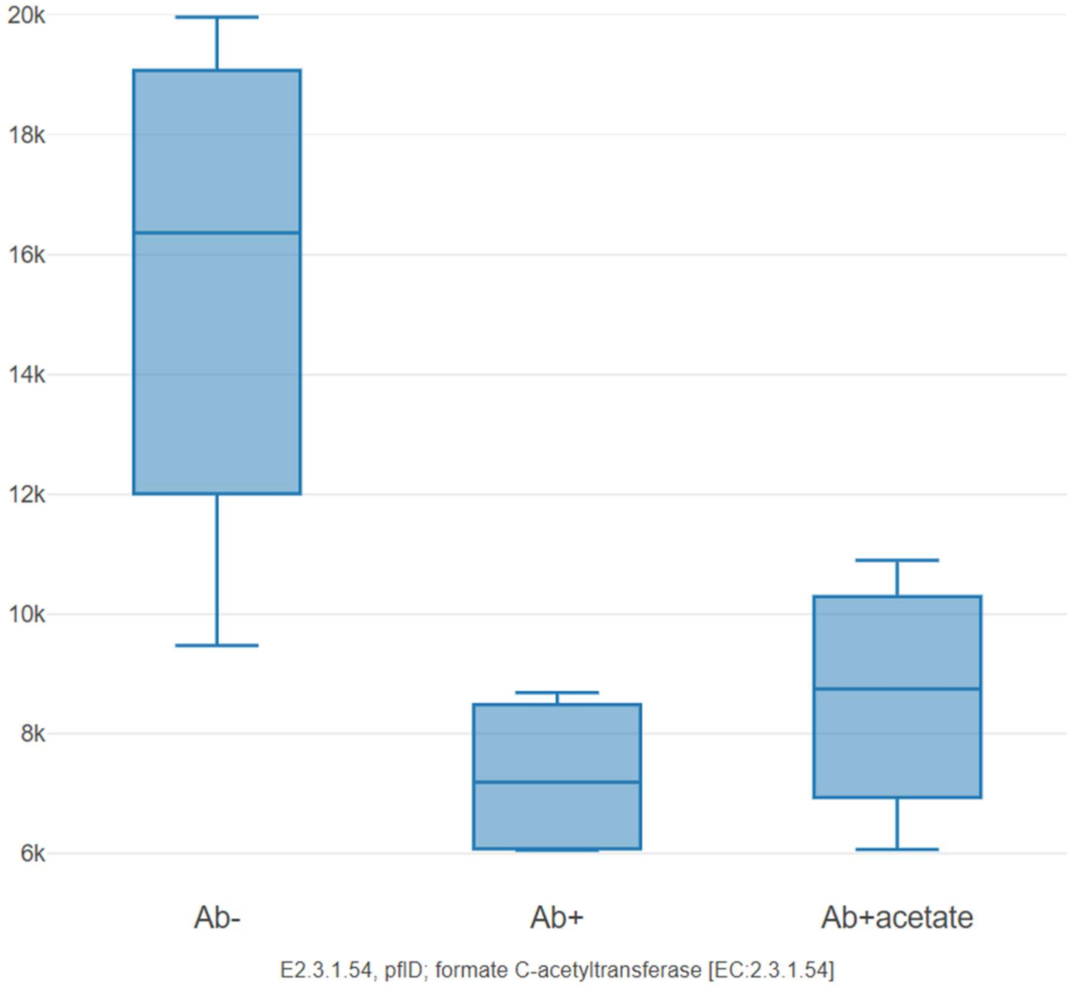
20k
18k
16k
14k
12k
10k
8k
6k
Ab-
Ab+
Ab+acetate
E2.3.1.54, pflD; formate C-acetyltransferase [EC:2.3.1.54]

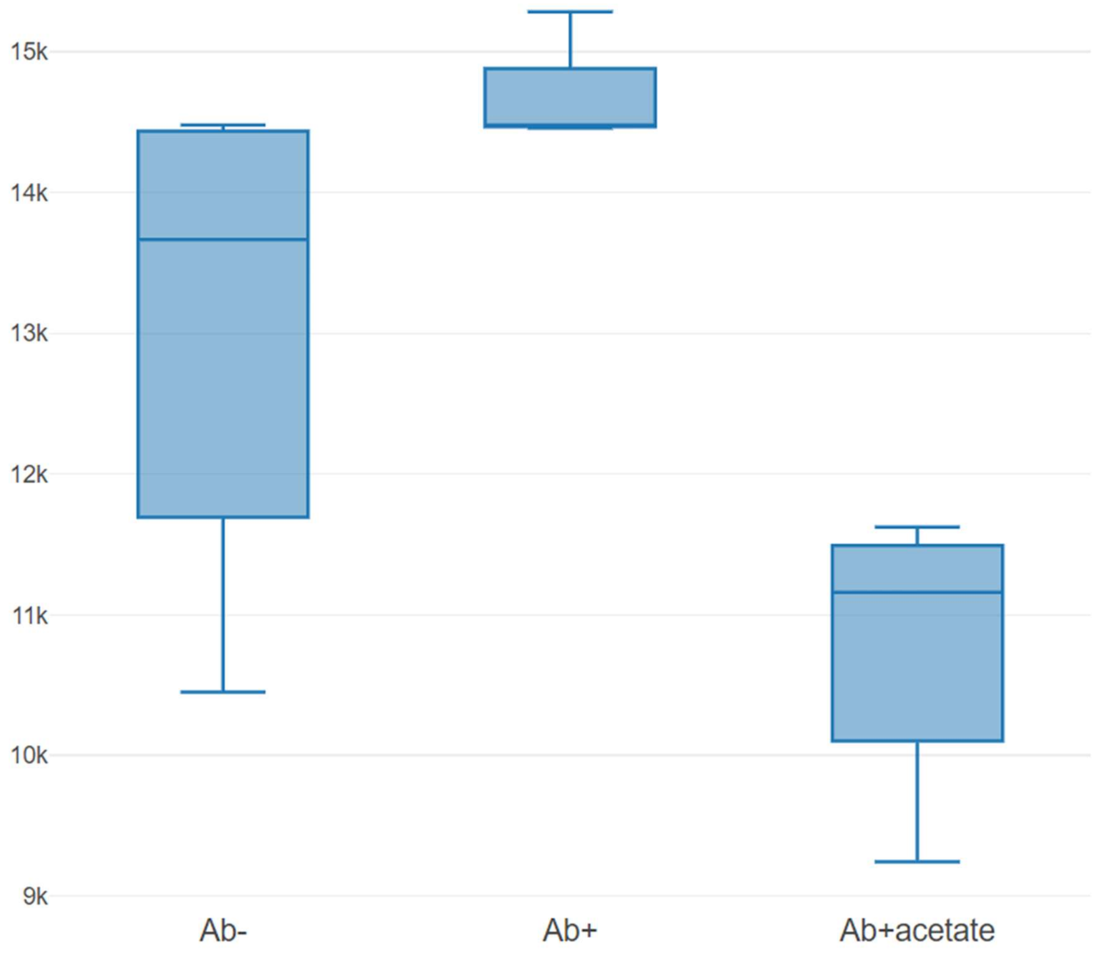

E2.3.1.8, pta; phosphate acetyltransferase [EC:2.3.1.8]

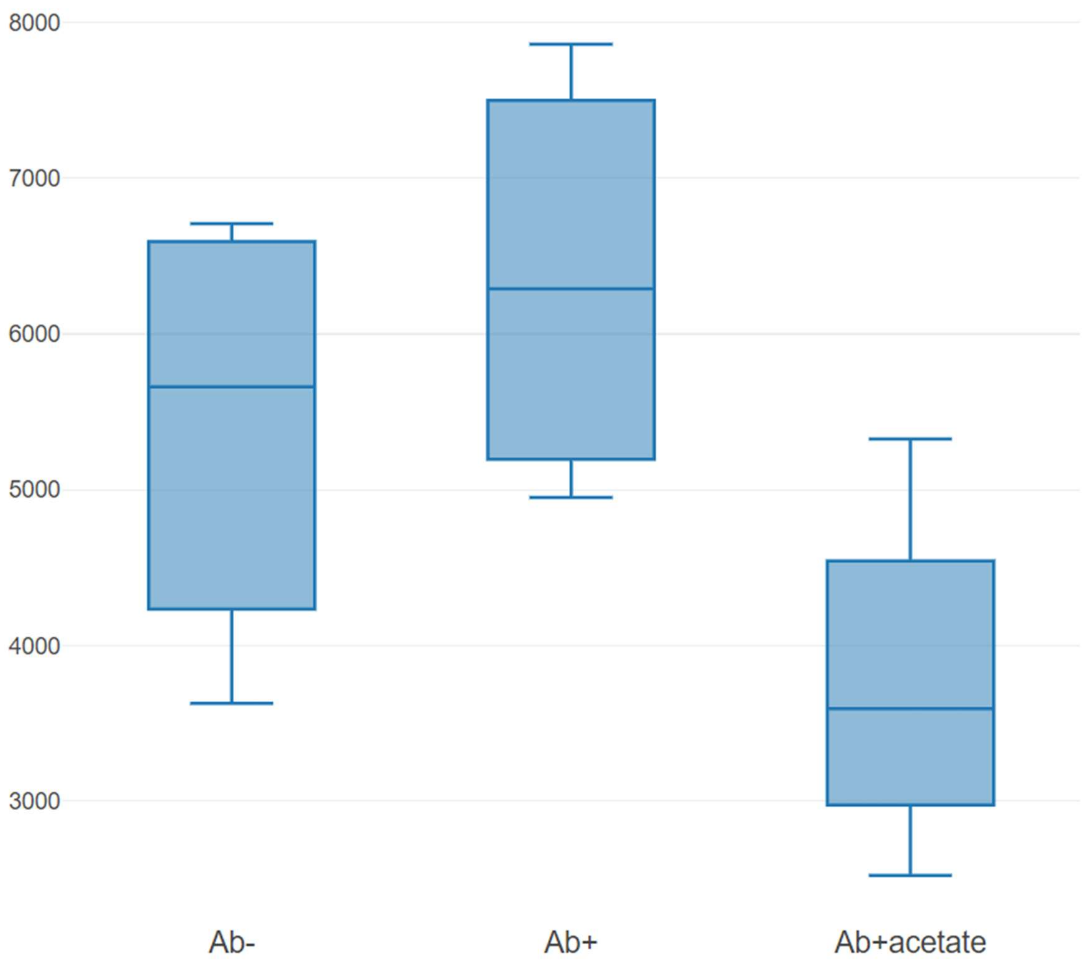

fucO; lactaldehyde reductase [EC:1.1.1.77]

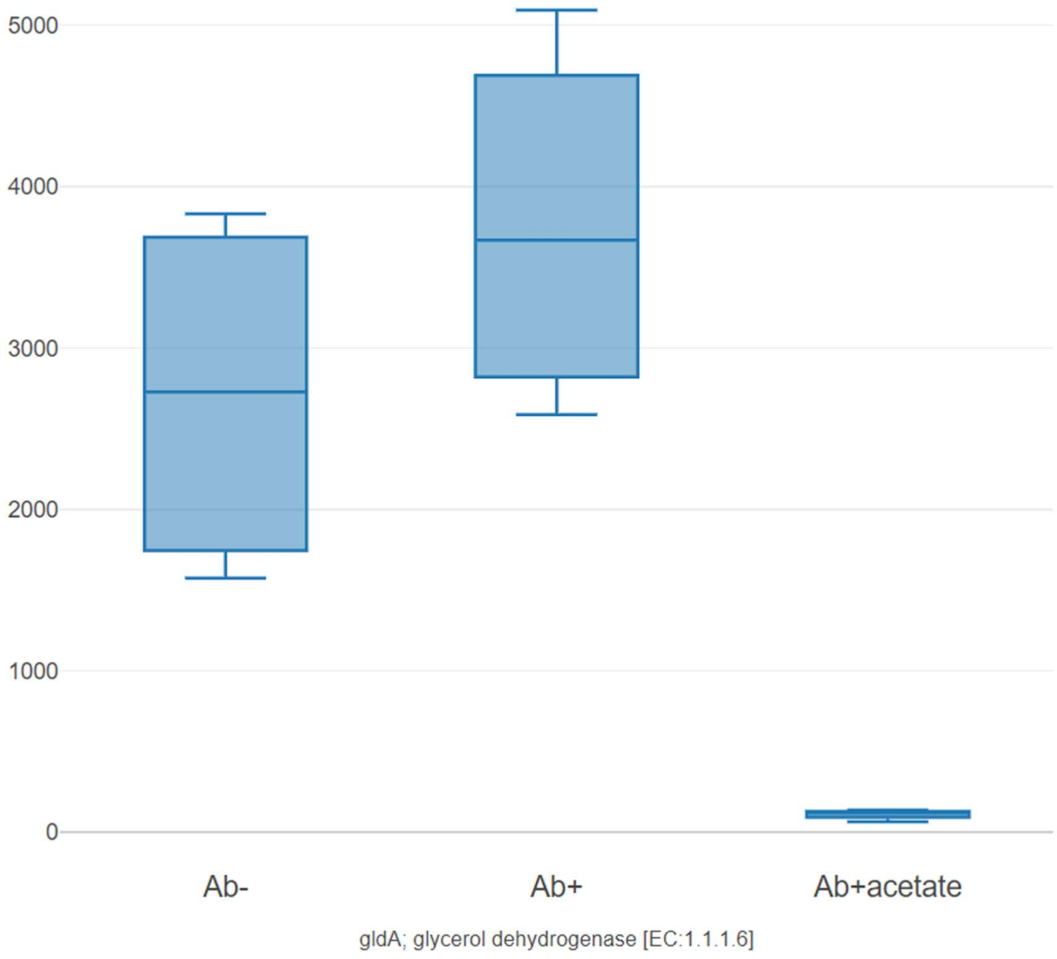
5000
4000
3000
2000
1000
0
Ab-
Ab+
Ab+acetate
gldA; glycerol dehydrogenase [EC:1.1.1.6]

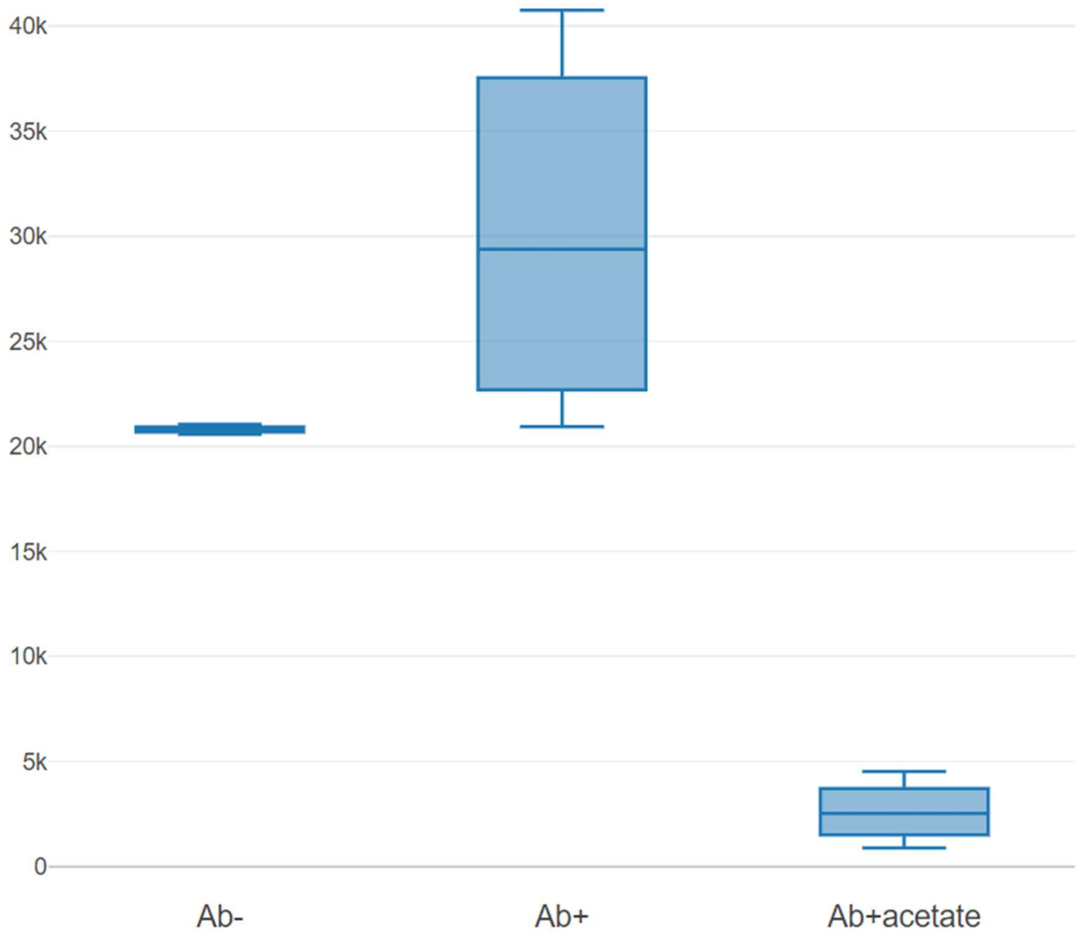

LDH, ldh; L-lactate dehydrogenase [EC:1.1.1.27]

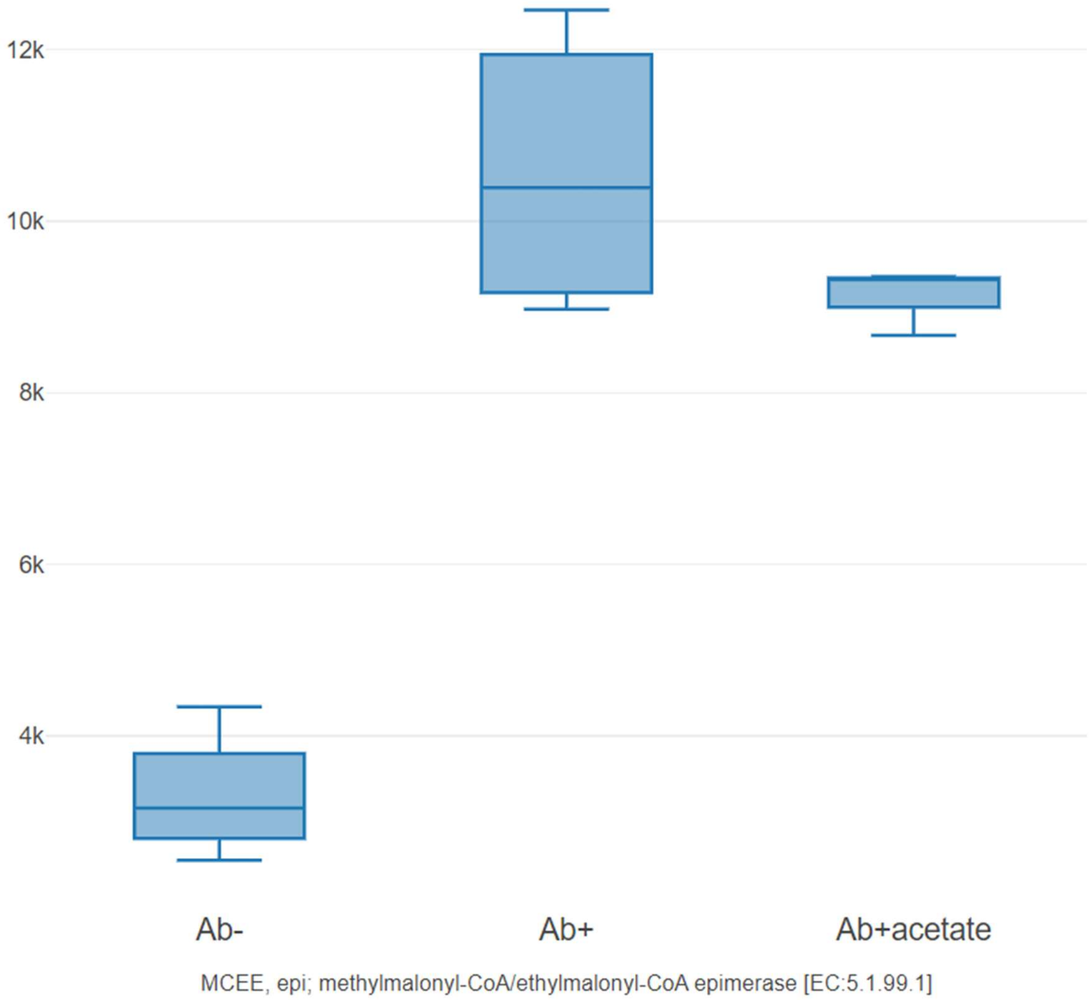
12k
10k
8k
6k
4k
Ab-
Ab+
Ab+acetate
MCEE, epi; methylmalonyl-CoA/ethylmalonyl-CoA epimerase [EC:5.1.99.1]

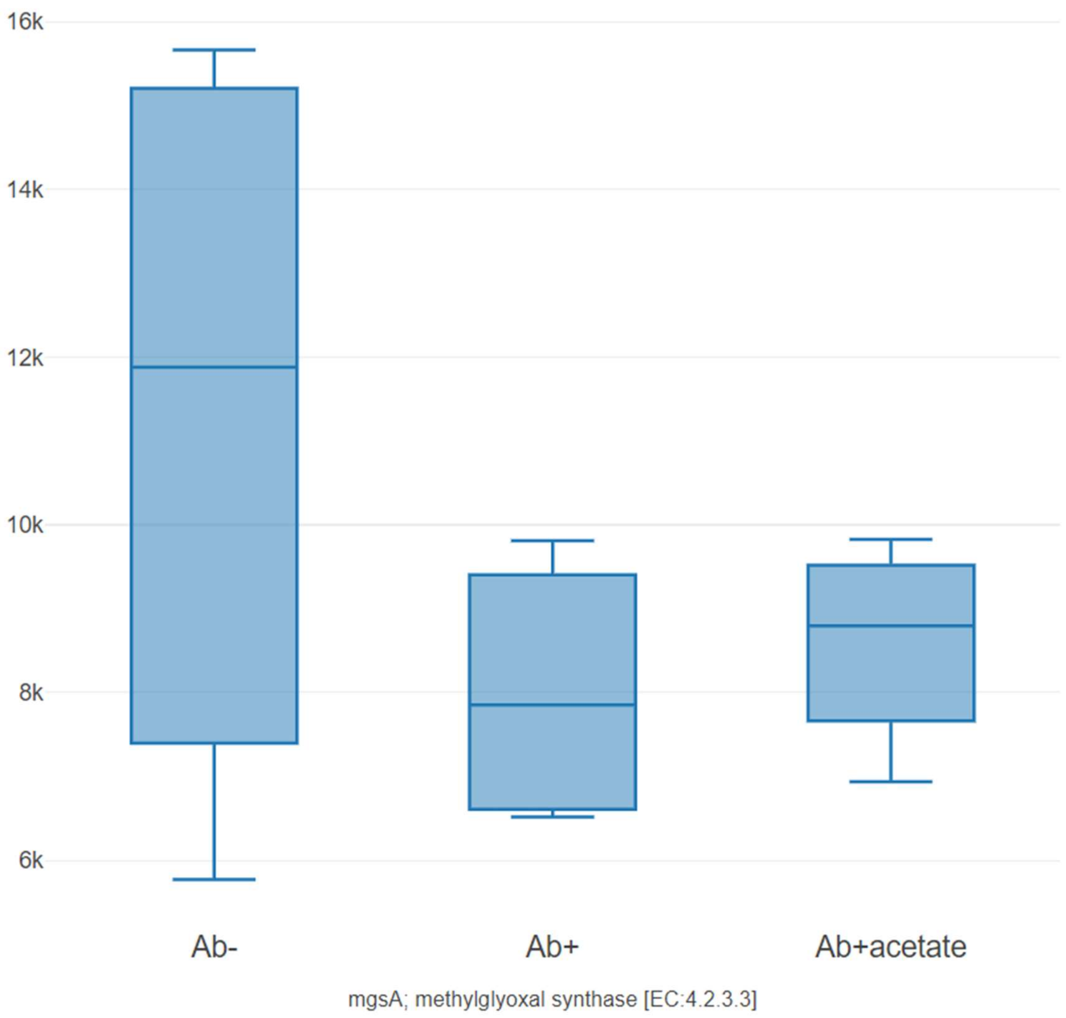
16k
14k
12k
10k
8k
6k
Ab-
Ab+
Ab+acetate
mgsA; methylglyoxal synthase [EC:4.2.3.3]

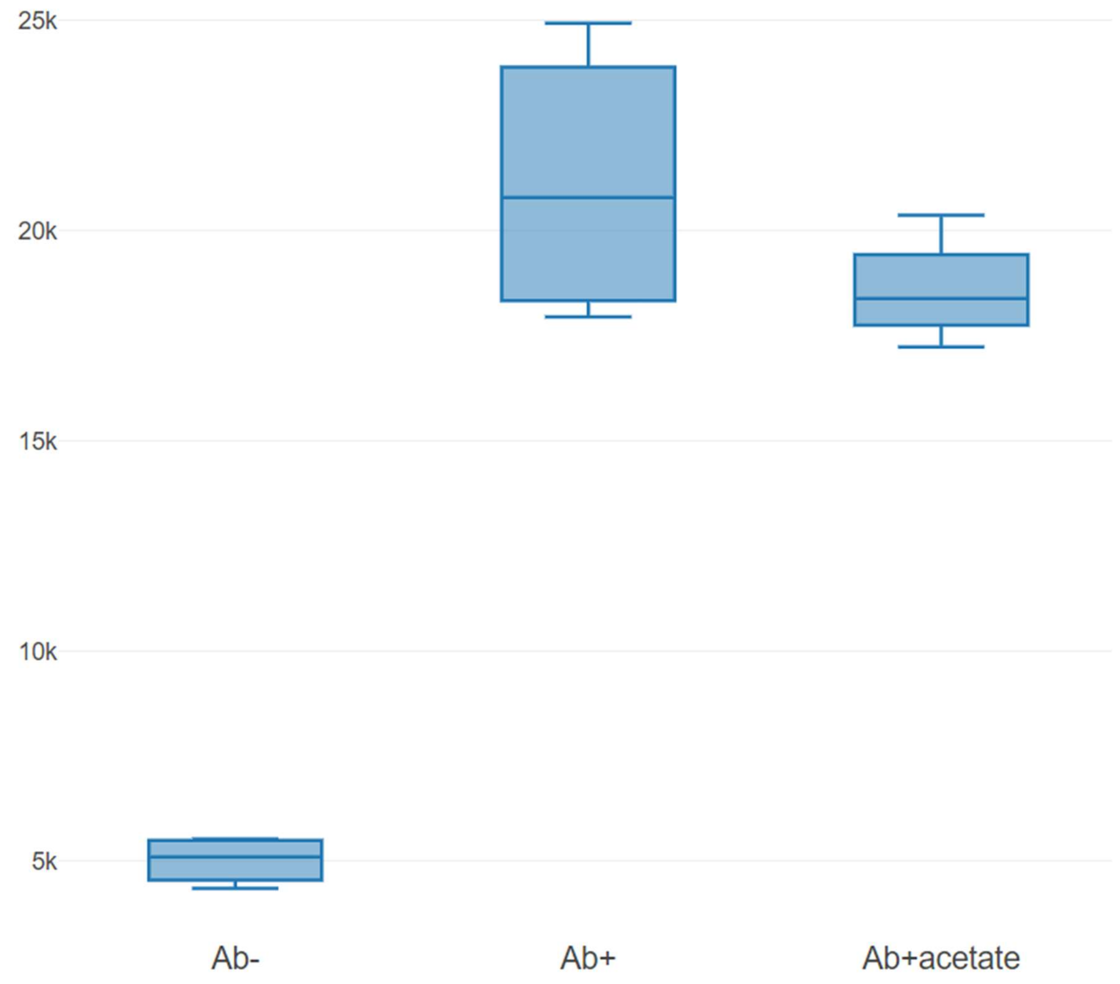

MUT; methylmalonyl-CoA mutase [EC:5.4.99.2]

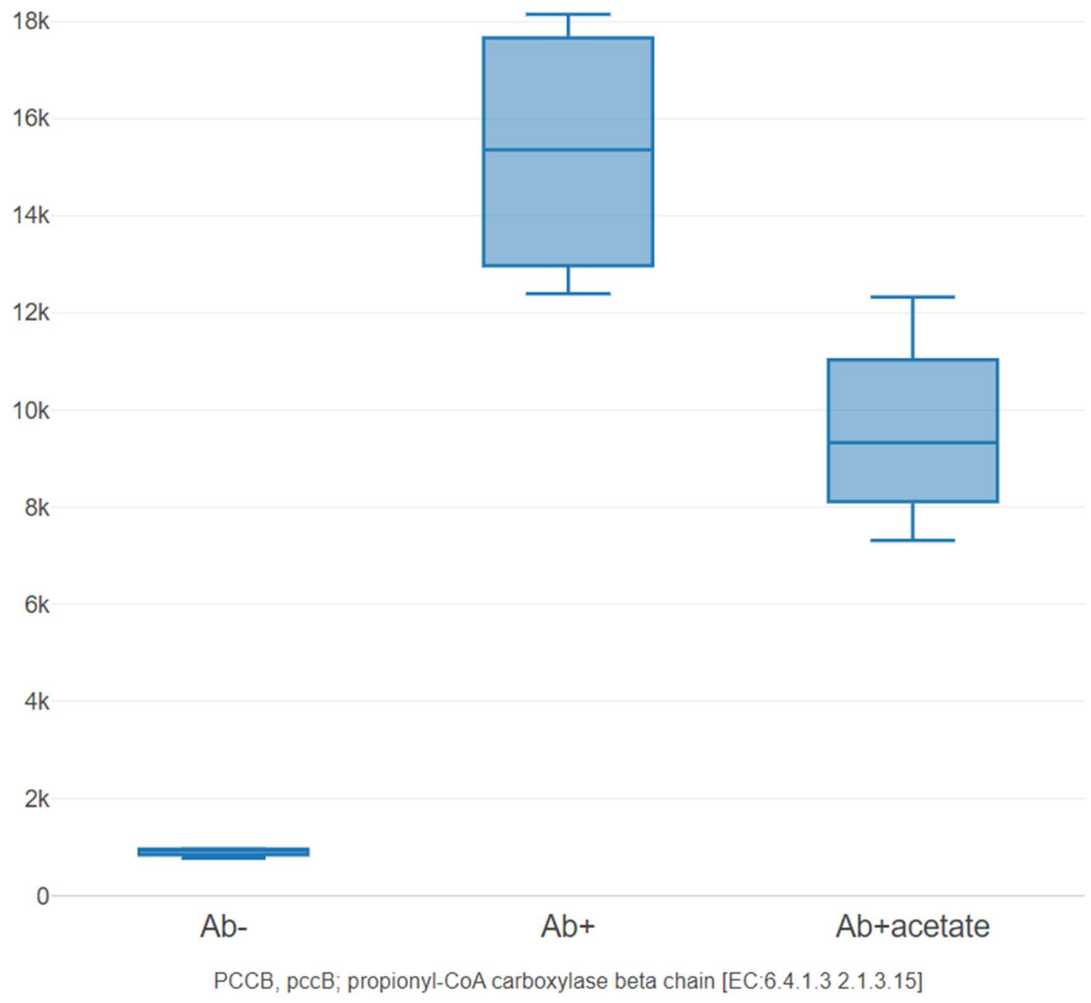
18k
16k
14k
12k
10k
8k
6k
4k
2k
0
Ab-
Ab+
Ab+acetate
PCCB, pccB; propionyl-CoA carboxylase beta chain [EC:6.4.1.3 2.1.3.15]

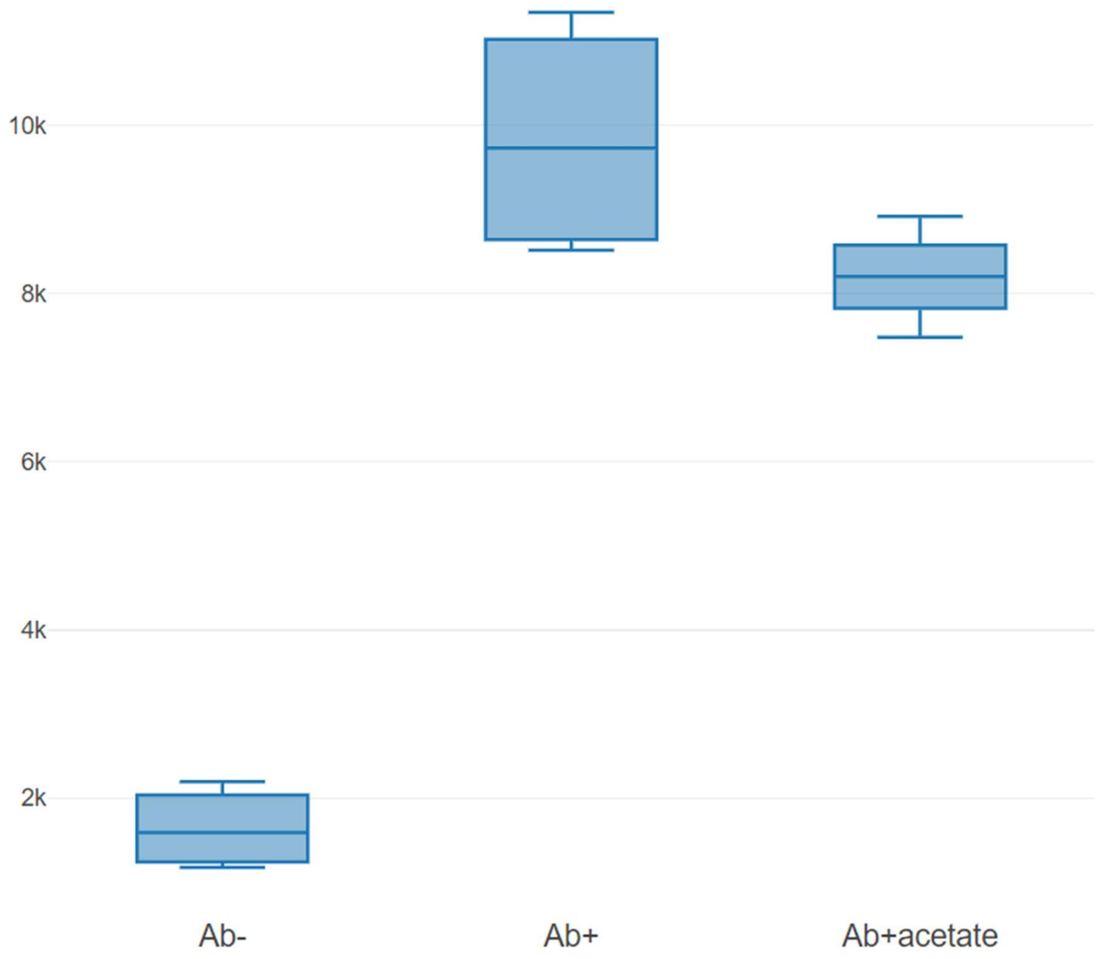

sucC; succinyl-CoA synthetase beta subunit [EC:6.2.1.5]

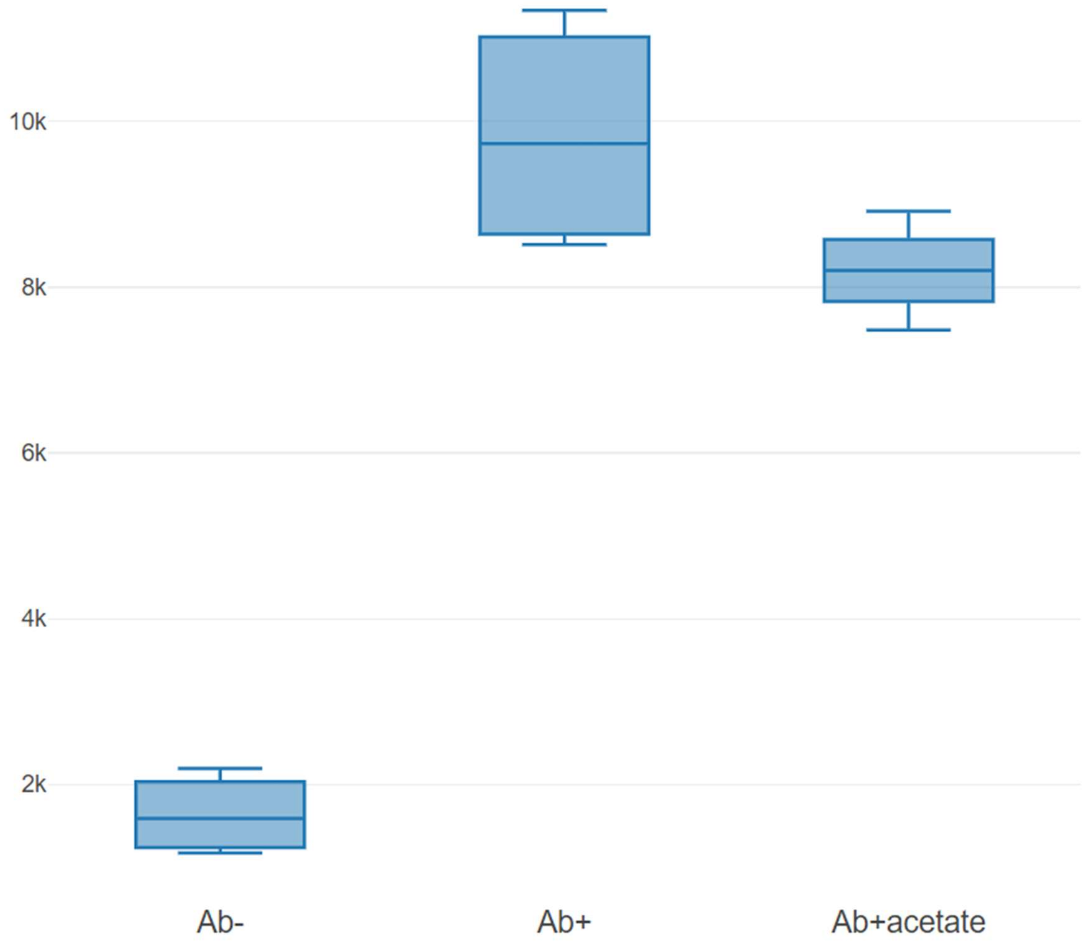

sucD; succinyl-CoA synthetase alpha subunit [EC:6.2.1.5]

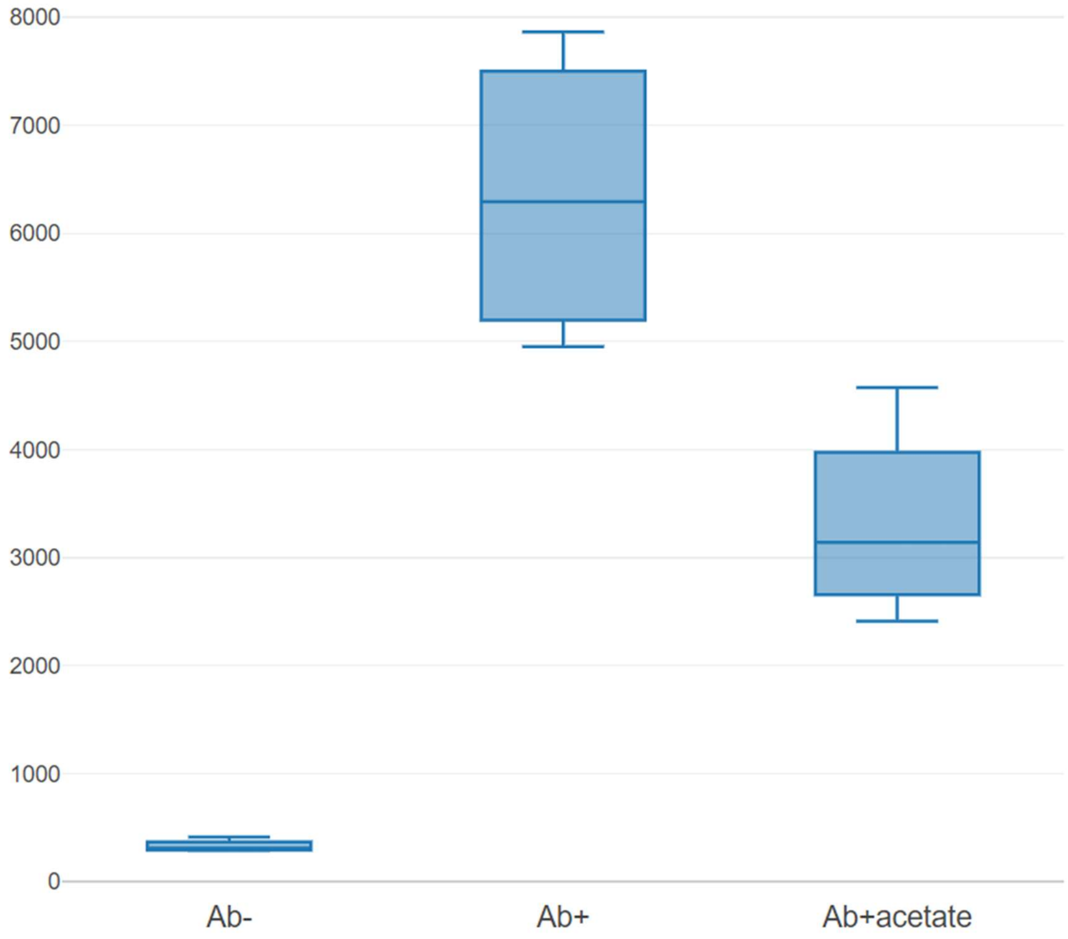

yqhD; NADP-dependent alcohol dehydrogenase [EC:1.1.-.-]
