# Supplementary material for: Acetate derived from the intestinal tract has a critical role in maintaining skeletal muscle mass and strength in mice
Source: Physiol Rep. 2024 Jun 4;12(11):e16047. doi: 10.14814/phy2.16047 (PMC11150057; doi:10.14814/phy2.16047)
Supplement: Supplementary file 5 — Figure S3: https://doi.org/10.6084/m9.figshare.25674135. Expression of proteasomal catabolic genes in the skeletal muscle during prolonged fasting. [file PHY2-12-e16047-s001.pdf]

Supplemental Figure S3

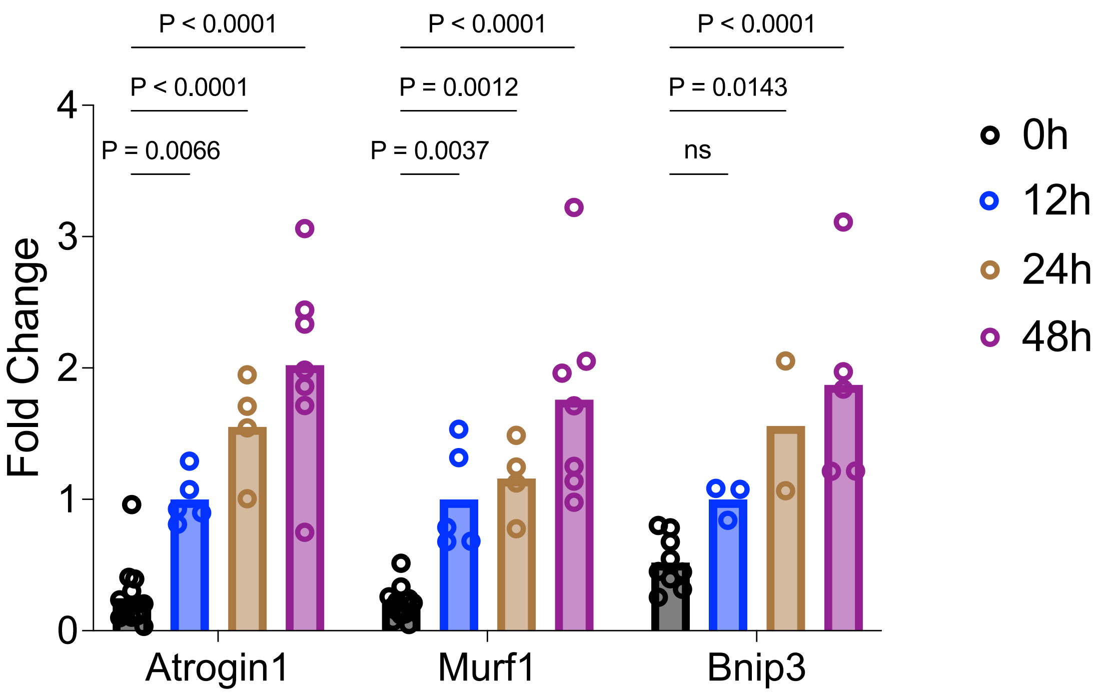

Supplemental Figure S3. Expression profile of genes related to skeletal muscle degradation in TA muscle. Quantitative PCR analysis of intracellular ubiquitin ligases-muscle atrophy F-box (Atrogin1 / MAFbx), muscle RING finger 1 (Murf1), and the autophagy-related gene, Bcl-2 and 19-kDa interacting protein 3 (Bnip3). TA muscles were harvested after 0h (Atrogin1, n=14; Murf1, n=14; Bnip3 n=9), 12h (Atrogin1, n=5; Murf1, n=5; Bnip3, n=3), 24h (Atrogin1, n=4; Murf1, n=4; Bnip3 n=3), or 48h (Atrogin1, n=7; Murf1, n=7; Bnip3, n=5). TA: tibialis anterior muscle. Data expressed as mean; ns, not statistically significant. Analyzed using, one-way ANOVA and subsequent post-hoc Tukey tests.
