# Supplementary material for: Acetate derived from the intestinal tract has a critical role in maintaining skeletal muscle mass and strength in mice
Source: Physiol Rep. 2024 Jun 4;12(11):e16047. doi: 10.14814/phy2.16047 (PMC11150057; doi:10.14814/phy2.16047)
Supplement: Supplementary file 6 — FIGURE S4: https://doi.org/10.6084/m9.figshare.25672272.v1. Skeletal muscle masses and Grip strength per bodyweight. [file PHY2-12-e16047-s003.pdf]

Supplemental Figure S4

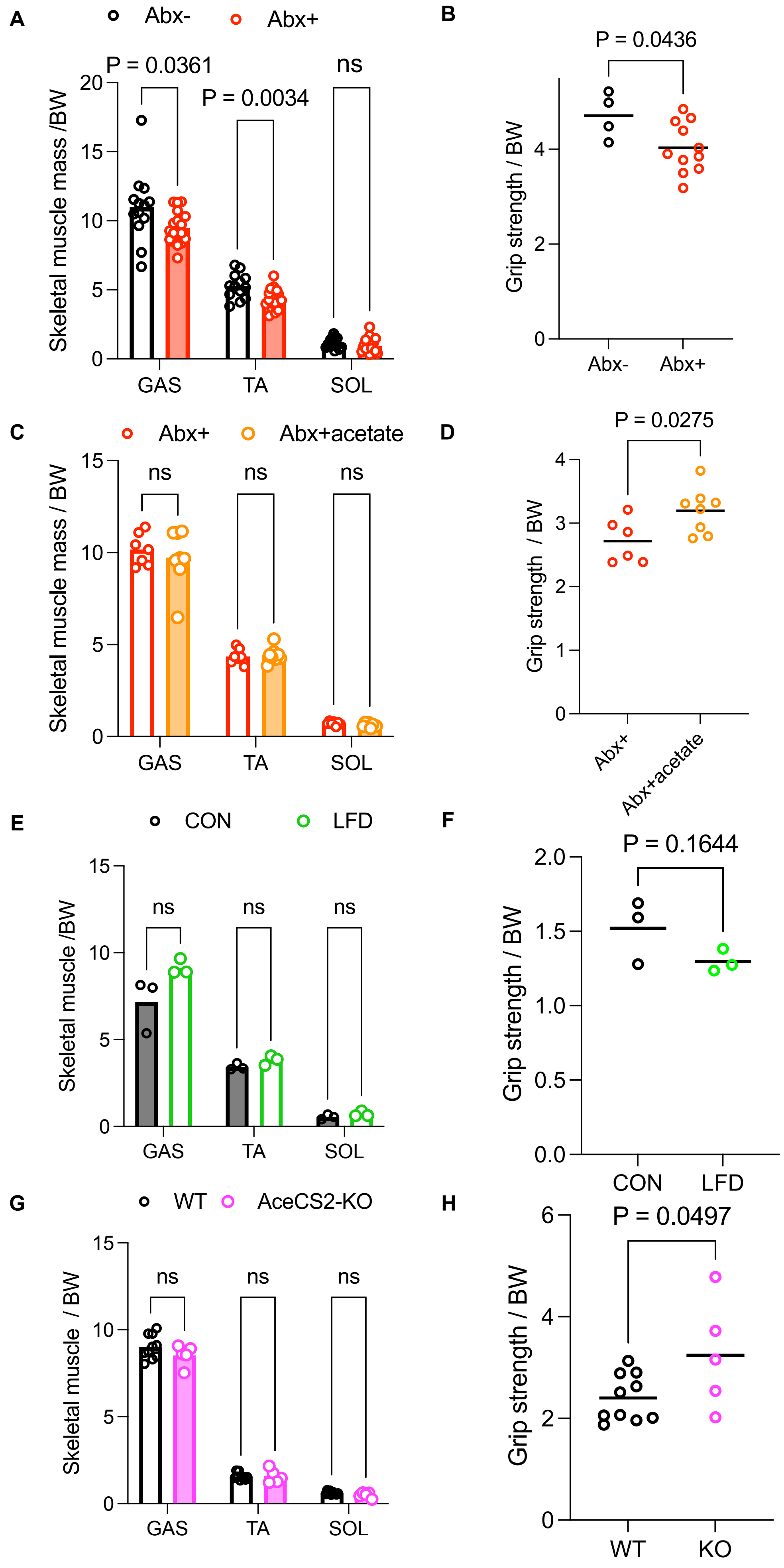

Supplemental Figure S4. *A*: Skeletal muscle masses / bodyweight of Abx- and Abx+ mice (Abx-, n=13; Abx+, n=18). *B*: Grip strength / bodyweight of Abx- and Abx+ mice (Abx-, n=4; Abx+, n=11). *C*: Skeletal muscle masses / bodyweight of Abx+ and Abx+ acetate mice (Abx+, n=7; Abx+ acetate, n=8). *D*: Grip strength / bodyweights of Abx+ and Abx+ acetate mice (Abx+, n=6; Abx+ acetate, n=8). *E*: Skeletal muscles / bodyweight of LFD and CON mice (LFD, n-3; CON, n=3). *F*: Grip strength / bodyweight of LFD and CON mice (LFD, n-3; CON, n=3). *G*: Skeletal muscle masses / grip strength of WT and AceCS2-KO mice (WT, n=10; AceCS2-KO, n=5). *H*: Grip strength / bodyweight of WT and AceCS2-KO mice (WT, n=10; AceCS2-KO, n=5). Data expressed as mean; ns, not statistically significant. The lines indicate the means; analyzed using Student’s t-tests. Abx-: antibiotics untreated, Abx+: antibiotics treated, Abx+ acetate+: antibiotics with acetate treated, GAS: gastrocnemius muscle, TA: tibialis anterior muscle, SOL: soleus muscle, LFD: low-fiber diet, CON: control diet.
